# Leading dietary determinants identified using machine learning techniques and a healthy diet score for changes in cardiometabolic risk factors in children: a longitudinal analysis

**DOI:** 10.1186/s12937-020-00611-2

**Published:** 2020-09-19

**Authors:** Xianwen Shang, Yanping Li, Haiquan Xu, Qian Zhang, Ailing Liu, Songming Du, Hongwei Guo, Guansheng Ma

**Affiliations:** 1 National Institute for Nutrition and Health, Chinese Center for Disease Control and Prevention, Beijing, China; 2 School of Behavioural and Health Sciences, Australian Catholic University, Melbourne, Australia; 3 Department of Medicine (Royal Melbourne Hospital), University of Melbourne, Melbourne, Australia; 4 Department of Nutrition, Harvard T. H. Chan School of Public Health, Boston, MA USA; 5 Institute of food and nutrition development, Ministry of Agriculture and Rural Affairs, Beijing, China; 6 Chinese Nutrition Society, Beijing, China; 7 School of Public Health, Fudan University, Shanghai, China; 8 Department of Nutrition and Food Hygiene, School of Public Health, Peking University, 38 Xue Yuan Road, Beijing, 100191 China

**Keywords:** Cardiometabolic risk factors, Leading dietary determinants, Healthy diet score, Machine learning, Children

## Abstract

**Background:**

Identifying leading dietary determinants for cardiometabolic risk (CMR) factors is urgent for prioritizing interventions in children. We aimed to identify leading dietary determinants for the change in CMR and create a healthy diet score (HDS) to predict CMR in children.

**Methods:**

We included 5676 children aged 6–13 years in the final analysis with physical examinations, blood tests, and diets assessed at baseline and one year later. CMR score (CMRS) was computed by summing Z-scores of waist circumference, an average of systolic and diastolic blood pressure (SBP and DBP), fasting glucose, high-density lipoprotein cholesterol (HDL-C, multiplying by − 1), and triglycerides. Machine learning was used to identify leading dietary determinants for CMR and an HDS was then computed.

**Results:**

The nine leading predictors for CMRS were refined grains, seafood, fried foods, sugar-sweetened beverages, wheat, red meat other than pork, rice, fungi and algae, and roots and tubers with the contribution ranging from 3.9 to 19.6% of the total variance. Diets high in seafood, rice, and red meat other than pork but low in other six food groups were associated with a favorable change in CMRS. The HDS was computed based on these nine dietary factors. Children with HDS ≥8 had a higher decrease in CMRS (β (95% CI): − 1.02 (− 1.31, − 0.73)), BMI (− 0.08 (− 0.16, − 0.00)), SBP (− 0.46 (− 0.58, − 0.34)), DBP (− 0.46 (− 0.58, − 0.34)), mean arterial pressure (− 0.50 (− 0.62, − 0.38)), fasting glucose (− 0.22 (− 0.32, − 0.11)), insulin (− 0.52 (− 0.71, − 0.32)), and HOMA-IR (− 0.55 (− 0.73, − 0.36)) compared to those with HDS ≦3. Improved HDS during follow-up was associated with favorable changes in CMRS, BMI, percent body fat, SBP, DBP, mean arterial pressure, HDL-C, fasting glucose, insulin, and HOMA-IR.

**Conclusion:**

Diets high in seafood, rice, and red meat other than pork and low in refined grains, fried foods, sugar-sweetened beverages, and wheat are leading healthy dietary factors for metabolic health in children. HDS is strongly predictive of CMR factors.

## Introduction

The pandemic and increasing trend of obesity-related cardiometabolic risk (CMR) factors are a public health challenge globally [1, 2]. Data from the China Health and Nutrition Survey (CHNS) in 2009 showed that there was a high prevalence of CMR factors in both children and adults [3], which imposes a tremendous burden on health care systems. Childhood CMR factors are highly likely to persist into adulthood and are associated with cardiovascular disease, diabetes, and mortality in the future [4–8]. Therefore, it is imperative to slow or reverse the increasing trend in the prevalence of CMR factors at an early stage of life [9, 10].

Diet is of paramount importance for the prevention of CMR factors [11]. Strong evidence from adults has shown that diets low in processed food, sugar-sweetened beverages (SSBs), and carbohydrate, and high in dairy and fish are associated with lower risks of cardiometabolic disorders including obesity, cardiovascular disease, diabetes, dyslipidemia, and hypertension [12, 13]. However, consumption of individual foods has not been demonstrated to be strongly predictive of CMR factors in children. A recent systematic review has shown that significant associations between dietary intakes and obesity-related CMR factors were observed in 19% of the 81 included studies in children [14]. Previous studies are also limited by small sample sizes, cross-sectional design, or failure to adjust for important confounders. Although dietary patterns have been well linked to CMR factors in children in some countries [15–17], they cannot be applied to other populations given that a healthy diet pattern in one study can be hardly derived from other studies. For example, a healthy diet pattern in one study was high in vegetables, fruits, and dairy [15], while a healthy pattern in another study was high in vegetables, fruits, fish, crackers, and bread [17]. Diet indices have also been developed for diet quality assessment, however, these indices are shown to be weak predictors of CMR factors [18, 19]. Identifying leading dietary determinants for changes in CMR factors using new methods is urgent for targeting intervention priorities for the prevention of CMR in children. It is also important to create a healthy diet score (HDS) to predict CMR factors in children.

We used machine learning techniques to identify leading dietary determinants for changes in CMR factors in children based on longitudinal data. We then created an HDS based on the identified leading determinants to predict CMR factors.

## Methods

### Participant selection

**Fig. 1:**
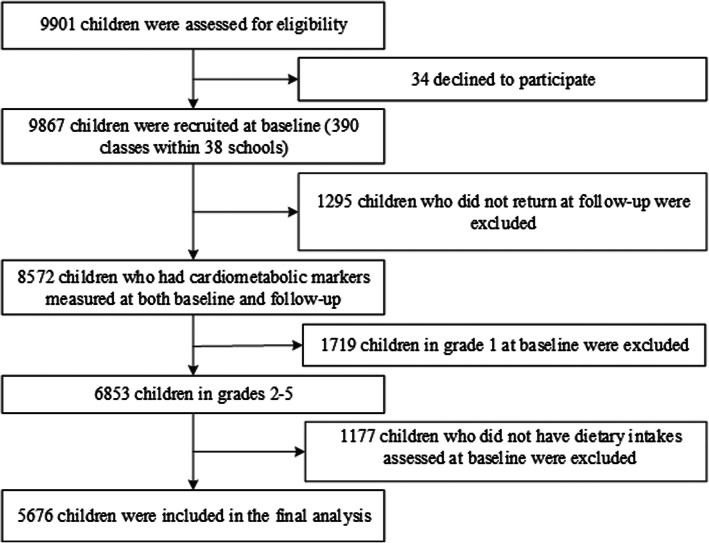
Flowchart for population section

The present analysis was based on a multicenter, randomized cluster controlled trial and the full description of the study has been published elsewhere [20]. Briefly, the study was conducted in six capital or province capital cities including Beijing, Shanghai, Chongqing, Jinan, Harbin, and Guangzhou. Data were collected at both baseline (May 2009) and follow-up (May 2010). Children in the intervention group received nutrition lectures (knowledge, attitudes, and dietary habits) as well as participated in two times of ten minutes or one time 20 min of Happy 10 program per day (involves various physical activities such as games, dances, and gymnastics, which were designed to stimulate children to enjoy physical activity). A total of 9901 children from 390 classes within 38 schools were screened for eligibility. Among 9867 children who were assessed at baseline, 8572 were reassessed at follow-up. Participants whose dietary intakes were not assessed, those who fell in the top (3500 Kcal/day) or bottom (300 Kcal/day) percentile of total energy intake, and who had missing values in all cardiometabolic measurements were excluded (*n* = 2896). A total of 5676 participants were included in the final analysis (Fig. 1).

The study protocol was approved by the Ethical Review Committee of the National Institute for Nutrition and Food Safety, Chinese Centre for Disease Control and Prevention. Written informed consent was obtained from the next of kin, carers, or guardians of all participants.

### Dietary assessment

Dietary intake was assessed using 24-h diet recalls for three consecutive days including two weekdays and one weekend day in children in grades 2–5. Interviews were conducted by trained investigators. During the interview, samples of local household dishes and utensils (different sizes of bowls, plates, and spoons) were displayed to the children. They were then shown pictures of common foods eaten in these dishes or utensils to indicate portion size consumed. The interviewer and the tutor would help children recall food intake at school while parents would help recall foods consumed at home.

A total of 1169 different food items were collected among all children and they were categorized into 26 groups according to nutrient contents as below: rice (boiled), wheat (such as steamed bun, noodles, and brans), refined grains (such as white breads, pizza, muffins, pancakes, and granola bar made by further processing grain powder), other cereals (such as corn, millet, and sorghum), fried foods, nuts and legumes (such as peanuts, walnuts, beans, and soybean products), starch roots and tubers, deep color vegetables, light color vegetables, edible fungi and algae (such as mushrooms, agaric, seaweed, and kelp), pickled vegetables (such as pickled mustard root, pickled sweet garlic, pickled cucumber, and pickled radish), fruits, pork, poultry, red meat other than pork (beef, lamb, other red meat), animal offal, processed meat (such as ham, beef jerky, and luncheon meat), seafood (such as fish, lobster, and crab), eggs, milk, yogurt, dairy product (such as milk powder and cheese), catsup and other sources, SSBs, candy and sugar, and dessert. Deep color vegetables were classified as carotene content ≥500 μg/100 g and light color vegetable with carotene < 500 μg/100 g. Nutrients and energy intake was calculated based on the China Food Composition [21]. The average amount of food and nutrient intake per day was calculated and energy-adjusted food and nutrient consumption were computed as ([100 × weight in grams]/total energy intake in Kcal).

### Confounders

Puberty status was recorded by investigators during the interview when physical examinations were conducted. Physical activity was assessed using a validated questionnaire in children, from which metabolic equivalent (MET) was calculated [22]. The questionnaire is a 39-item self-administered questionnaire that captures the number of days in the past week, times per day, and the number of minutes per time engaging in the physical activity. MET was calculated according to the assigned metabolic values for each specified physical activity. Birthweight, household income, parental education, and parental height and weight were reported by parents using a self-administered questionnaire.

### Physical examinations and blood tests

Physical examinations and blood tests (10–14 h fasting beforehand) were performed at both baseline and follow-up following standardized procedures.

Height was measured to the nearest 0.1 cm and weight to the nearest 0.1 kg. Body mass index (BMI) was computed as weight in kilograms divided by the square of height in meters. Waist circumference (WC) was measured midway between the lowest rib and the superior border of the iliac crest on expiration to the nearest 0.1 cm and the average of two measurements was used.

Body composition was assessed using a single frequency (50 Hz) hand to foot bioelectrical impendence device (ImpDF50, Impedimed Pty Ltd., Qld, Australia). Body fat mass was computed using the prediction formula developed by Deurenberg et al. [23] and percent body fat (PBF) was calculated as fat mass divided by body weight.

Blood pressure was measured in the seated position using a mercury sphygmomanometer (XJ300/40–1, Made in Shanghai) by trained nurses with at least 10 min rest before the measurement. The first and the fifth Korotkoff sounds were used to represent the systolic and diastolic blood pressure (SBP and DBP). Three measurements were taken to the nearest two mmHg and the average of the last two measurements was used. Mean arterial pressure (MAP) was calculated as (DBP + 0.33 × [SBP-DBP]).

Fasting glucose was measured using the glucose-oxidize method (Daiichi Pharmaceutical Co., Ltd., Tokyo, Japan) within four hours after the fasting blood sample was obtained. Fasting insulin was measured using the immunoenzymatic method (analyzer AXSYM, Abbott Co., Ltd., Japan). The homeostatic model assessment of insulin resistance (HOMA-IR) was computed as (fasting insulin [μU/L] × fasting glucose [mg/dL])/405.

Conventional enzymatic assays were used to measure levels of serum triglycerides (TG), total cholesterol (TC), high-density lipoprotein cholesterol (HDL-C), low-density lipoprotein cholesterol (LDL-C) with 7080 Automatic Analyzer (Daiichi Pharmaceutical Co., Ltd., Tokyo, Japan).

### Statistical analysis

BMI, WC, PBF, SBP, DBP, MAP, TG, TC, HDL-C, LDL-C, TG to HDL-C ratio, fasting glucose, insulin, and HOMA-IR were standardized (i.e. Z scores were calculated: Z = (value−mean)/SD using sex- and age-specific means and SDs). CMR score (CMRS) was calculated by summing Z scores of WC, the average of SBP and DBP, fasting glucose, HDL-C (multiplying by − 1), and TG [24].

We randomly selected 50% of all participants in the intervention study as training data and the remaining as testing data. We used three established machine learning models including multiple linear regression model, random forest, and gradient boost machine (GBM) to analyze the importance of 26 dietary predictors (baseline) for the change in CMRS based on the training data and compared the performance of these models based on the testing data. For multiple linear regression model, we selected Gaussian family distribution when established prediction model using machine learning techniques. The hyper-parameters alpha and lambda specify the regularization strength and the regularization distribution between L1 (LASSO) and (ridge regression) L2 penalties, respectively. The random forest algorithm is a supervised learning algorithm constructing an ensemble of decision-trees using randomly bootstrapping sample datasets and averaging predictions of its trees [25]. It applies a bagging method to ensemble multiple decision trees generated from subsets to reduce correlations among the constitute decision trees. In this study, we used the R-square to determine the best predicting variable and location for each tree split in our algorithm. We grew the forest with 500 trees and implemented a grid search to obtain optimal parameters including the number of variables randomly sampled as candidates at each split and the max depth of each tree (effectively the number of interactions are considered in the model) for the random forest.

GBM belongs to a family of machine learning approaches leveraging a boosting ensemble method. An ensemble of decision-trees was constructed using a weighted average of trees with more weight to those with a better performance [26]. GBM converts a weak original learning algorithm to a strong one by minimizing an exponential loss of the misclassification rate. A forest of 500 trees was applied and a grid search for model optimization was also conducted with the maximum number of models, the max depth of each tree, learning rate, row sample rate per tree, and column sample rate as hyper-parameters. Five-Fold cross-validation was applied to test if the models were overfitting. Regularization was conducted, and optimal parameters were used in modeling (Table S1). We realized these modeling exercises using the statistical software R 3.4.1 (toolbox h2o). Leading dietary factors were obtained according to their contribution derived from the machine learning method with the best performance.

A healthy diet score (HDS) was computed by summing sub-scores with each of the leading dietary predictors as one point according to their associations with CMRS. For example, more than the median intake of fruit was scored as 1 and equal or less as 0, if fruit intake was inversely associated with CMRS. We also calculated a HDS by summing weighted sub-scores according to the contribution of the corresponding dietary predictors derived from the machine learning method. ANOVA for continuous variables and Chi-square tests for categorical variables were performed to compare the difference of baseline characteristics across HDS.

Since the interaction between intervention/sex and HDS for changes in most CMR factors was not significant (Table S2 and S3), we did the analysis for the association between HDS and CMR factors in the whole population.

The general linear regression model (GLM) was used to test the difference in changes in CMR factors between participants with different HDS. We tested the following models: 1) classes in schools were adjusted for as random effects and characteristics of the individuals including age, sex, and corresponding CMR factor at baseline as fixed effects; 2) model 1 plus intervention group, grade, puberty, BMI, physical activity, and intake of energy, fiber, vegetable, fruit, pork, legumes, and nuts at baseline; 3) model 2 plus birth weight, breastfeeding, household income, parental BMI and education. We used the Benjamin-Hochberg procedure to control the false discovery rate at level of 5% for multiple comparisons [27]. Bonferroni *P*-value adjustments were performed for all pairwise comparisons. The association between change in HDS and changes in CMR factors was also tested using GLM. Changes in CMR factors were calculated by subtracting the results at baseline from those at follow-up. HDS at follow-up was calculated based on the nine leading dietary determinants and improved HDS referred to an increase in HDS (subtracting HDS at baseline from that at follow-up). For individual CMR factors, a standardized mean difference of 0.2, 0.5, and ≥ 0.8 represents a small, medium, and large effect size, respectively. As CMRS is the summing of Z-scores of five components, a standardized mean difference of 1.0, 2.5, and ≥ 4.0 represents a small, medium, and large effect size, respectively [28].

We did an interaction analysis to examine whether the association between HDS and CMRS was modified by sex, grade, birthweight, household income, parental BMI, and parental education.

We repeated the analysis for the association between HDS and changes in CMR factors in children in the control group. We also did external validation of our HDS in children aged 6–13 years from CHNS with diet and physical examinations measured in two or more surveys.

Analyses except modeling machine learning were performed using SAS version 9.4 (SAS Institute Inc.) and all *P* values were two-sided.

## Results

We included 5676 children (50.5% girls) aged 6–13 years (mean ± SD: 9.54 ± 1.19) in the final analysis. HDS was inversely associated with age, BMI, WC, PBF, and DBP at baseline and positively associated with TC, HDL-C, and LDL-C at baseline. There was not a significant association of HDS with CMRS at baseline. Higher HDS was associated with lower intake of energy, carbohydrate, fat, fiber, and iron and higher intake of protein, vitamin C, vitamin E, and carotene (Table 1).

**Table 1:** Baseline characteristics by healthy diet score

	Healthy Diet Score*						*P* value^†^
	≤3 (*n* = 861)	4 (*n* = 1021)	5 (*n* = 1328)	6 (*n* = 1420)	7 (*n* = 773)	≥8 (*n* = 299)	
Age (years)	9.75 ± 1.29^‡^	9.57 ± 1.18	9.49 ± 1.15	9.44 ± 1.19	9.56 ± 1.11	9.47 ± 1.14	< 0.0001
BMI (kg/m^2^)	17.50 ± 3.55	17.23 ± 3.21	17.31 ± 3.20	17.04 ± 3.09	17.02 ± 2.93	16.79 ± 2.87	< 0.0001
WC (cm)	59.30 ± 9.50	58.94 ± 9.32	58.67 ± 8.76	57.83 ± 8.61	58.04 ± 8.19	57.49 ± 8.07	< 0.0001
PBF (%)	24.65 ± 4.65	24.32 ± 4.66	24.22 ± 4.91	23.53 ± 4.92	23.26 ± 4.84	23.01 ± 4.72	< 0.0001
SBP (mm Hg)	101.21 ± 11.09	100.92 ± 10.80	100.37 ± 10.89	100.24 ± 10.98	100.54 ± 10.61	100.75 ± 10.14	0.11
DBP (mm Hg)	64.91 ± 9.31	64.23 ± 9.38	64.18 ± 8.89	63.81 ± 9.09	63.89 ± 8.56	64.00 ± 8.89	0.013
TC (mmol/L)	3.96 ± 0.71	3.97 ± 0.73	4.04 ± 0.77	4.15 ± 0.83	4.25 ± 0.83	4.28 ± 0.77	< 0.0001
HDL-C (mmol/L)	1.46 ± 0.31	1.46 ± 0.32	1.45 ± 0.29	1.48 ± 0.30	1.51 ± 0.30	1.49 ± 0.30	0.0012
LDL-C (mmol/L)	1.94 ± 0.64	2.02 ± 0.60	2.14 ± 0.64	2.20 ± 0.63	2.26 ± 0.63	2.27 ± 0.58	< 0.0001
TG (mmol/L)	0.79 ± 0.43	0.80 ± 0.41	0.85 ± 0.48	0.84 ± 0.46	0.82 ± 0.45	0.78 ± 0.39	0.18
Fasting glucose (mmol/L)	4.47 ± 0.61	4.46 ± 0.60	4.48 ± 0.58	4.55 ± 0.53	4.61 ± 0.47	4.62 ± 0.42	< 0.0001
CMRS	−0.27 ± 2.46	−0.25 ± 2.53	−0.09 ± 2.35	−0.26 ± 2.34	−0.30 ± 2.30	−0.22 ± 2.35	0.86
Physical activity (MET/week)	608.3 ± 444.3	621.4 ± 490. 8	666.3 ± 592.7	620.0 ± 586.1	605.8 ± 613.0	676.4 ± 723.9	0.55
Energy (kcal/day)	1268.6 ± 529.1	1281.9 ± 594.2	1326.8 ± 626.0	1274.8 ± 582.0	1217.1 ± 560.3	1152.5 ± 523.4	0.0018
Refined grains (gram/100 kcal/day)	1.60 ± 2.54	1.62 ± 3.63	1.93 ± 4.40	3.21 ± 6.18	3.64 ± 6.12	3.39 ± 6.22	< 0.0001
Seafood (gram/100 kcal/day)	0.44 ± 1.61	0.96 ± 2.37	1.56 ± 2.93	2.72 ± 3.90	4.19 ± 4.97	5.17 ± 4.86	< 0.0001
Fried foods (gram/100 kcal/day)	1.96 ± 2.37	1.15 ± 2.11	0.66 ± 1.85	0.28 ± 1.16	0.12 ± 0.62	0.04 ± 0.43	< 0.0001
Sugar-sweetened beverages (gram/100 kcal/day)	3.39 ± 5.07	2.84 ± 5.37	1.85 ± 4.47	1.28 ± 4.14	0.58 ± 2.48	0.23 ± 2.09	< 0.0001
Rice (gram/100 kcal/day)	3.97 ± 2.82	4.98 ± 4.19	6.32 ± 5.08	9.08 ± 6.73	11.21 ± 8.67	12.60 ± 6.61	< 0.0001
Wheat (gram/100 kcal/day)	7.28 ± 3.99	6.98 ± 4.48	6.41 ± 5.06	5.03 ± 4.91	3.47 ± 3.79	2.33 ± 2.44	< 0.0001
Fungi and algae (gram/100 kcal/day)	0.95 ± 1.32	0.69 ± 1.29	0.45 ± 1.25	0.32 ± 0.85	0.21 ± 0.77	0.08 ± 0.37	< 0.0001
Roots and tubers (gram/100 kcal/day)	2.76 ± 2.62	2.30 ± 3.01	1.71 ± 2.89	1.19 ± 2.56	0.72 ± 1.92	0.39 ± 1.23	< 0.0001
Red meat other than pork (gram/100 kcal/day)	0.24 ± 0.94	0.41 ± 1.27	0.67 ± 1.88	1.00 ± 2.29	1.30 ± 2.31	2.06 ± 2.95	< 0.0001
Protein intake (g/100 Kcal/day)	3.97 ± 0.81	4.10 ± 0.97	4.21 ± 1.04	4.39 ± 1.19	4.82 ± 1.22	5.17 ± 1.38	< 0.0001
Fat intake (g/100 Kcal/day)	3.03 ± 1.07	3.03 ± 1.16	2.92 ± 1.23	2.92 ± 1.21	2.93 ± 1.07	2.92 ± 0.99	0.0084
Carbohydrate intake (g/100 Kcal/day)	14.40 ± 2.58	14.24 ± 2.83	14.35 ± 3.07	14.20 ± 3.13	13.74 ± 2.90	13.35 ± 2.93	< 0.0001
Fibre intake (g/100 Kcal/day)	0.65 ± 0.36	0.58 ± 0.39	0.52 ± 0.32	0.48 ± 0.30	0.46 ± 0.25	0.43 ± 0.23	< 0.0001
Vitamin C intake (mg/100 Kcal/day)	3.15 ± 2.11	3.23 ± 2.58	3.11 ± 2.55	3.27 ± 2.88	3.45 ± 2.69	3.41 ± 2.57	0.0164
Vitamin E intake (mg/100 Kcal/day)	0.31 ± 0.26	0.29 ± 0.21	0.26 ± 0.16	0.26 ± 0.17	0.25 ± 0.15	0.26 ± 0.16	< 0.0001
Carotene intake (ug/100 Kcal/day)	73.42 ± 64.62	73.52 ± 83.18	72.64 ± 79.47	79.83 ± 95.19	83.16 ± 99.95	78.82 ± 82.90	0.0055
Magnesium intake (mg/100 Kcal/day)	15.19 ± 3.51	14.94 ± 3.59	14.77 ± 3.78	14.72 ± 3.77	15.12 ± 3.91	15.44 ± 4.15	0.99
Potassium intake (mg/100 Kcal/day)	102.00 ± 26.22	100.95 ± 32.25	97.17 ± 31.47	99.02 ± 35.06	102.44 ± 33.10	104.67 ± 32.89	0.55
Phosphorus intake (mg/100 Kcal/day)	58.39 ± 10.29	59.32 ± 12.05	60.12 ± 13.49	62.13 ± 15.07	65.51 ± 14.03	68.70 ± 16.19	< 0.0001
Calcium intake (mg/100 Kcal/day)	30.84 ± 13.12	30.61 ± 14.84	29.41 ± 15.20	30.01 ± 18.41	30.53 ± 15.54	31.44 ± 15.90	0.90
Iron intake (mg/100 Kcal/day)	1.39 ± 1.05	1.24 ± 0.88	1.14 ± 0.54	1.14 ± 0.52	1.14 ± 0.35	1.16 ± 0.29	< 0.0001
Sex							0.0001
Boys	384 (44.6)^§^	481 (47.1)	654 (49.2)	708 (49.9)	414 (53.6)	155 (51.8)	
Girls	477 (55.4)	540 (52.9)	674 (50.8)	712 (50.1)	359 (46.4)	144 (48.2)	
Grade							0.0001
Two	217 (25.2)	276 (27.0)	385 (29.0)	431 (30.4)	212 (27.4)	96 (32.1)	
Three	209 (24.3)	291 (28.5)	381 (28.7)	380 (26.8)	205 (26.5)	73 (24.4)	
Four	231 (26.8)	267 (26.2)	362 (27.3)	384 (27.0)	231 (29.9)	88 (29.4)	
Five	204 (23.7)	187 (18.3)	200 (15.1)	225 (15.8)	125 (16.2)	42 (14.0)	
Puberty							0.12
Yes	782 (90.8)	931 (91.2)	1239 (93.3)	1316 (92.7)	711 (92.0)	278 (93.0)	
No	79 (9.2)	90 (8.8)	89 (6.7)	104 (7.3)	62 (8.0)	21 (7.0)	
Birth weight							0.42
< 2500 g	31 (3.6)	27 (2.6)	50 (3.8)	56 (3.9)	23 (3.0)	10 (3.3)	
2500–3999 g	696 (80.8)	793 (77.7)	1048 (78.9)	1114 (78.5)	635 (82.1)	244 (81.6)	
≥ 4000 g	77 (8.9)	118 (11.6)	129 (9.7)	119 (8.4)	47 (6.1)	21 (7.0)	
Missing	57 (6.6)	83 (8.1)	101 (7.6)	131 (9.2)	68 (8.8)	24 (8.0)	
Mother’s BMI							0.0008
< 24 kg/m^2^	641 (74.4)	765 (74.9)	980 (73.8)	1113 (78.4)	587 (75.9)	258 (86.3)	
24–27.9 kg/m^2^	159 (18.5)	176 (17.2)	235 (17.7)	203 (14.3)	136 (17.6)	25 (8.4)	
≥ 28 kg/m^2^	24 (2.8)	28 (2.7)	40 (3.0)	22 (1.5)	15 (1.9)	6 (2.0)	
Missing	37 (4.3)	52 (5.1)	73 (5.5)	82 (5.8)	35 (4.5)	10 (3.3)	
Father’s BMI							< 0.0001
< 24 kg/m^2^	433 (50.3)	515 (50.4)	641 (48.3)	769 (54.2)	459 (59.4)	173 (57.9)	
24–27.9 kg/m^2^	307 (35.7)	346 (33.9)	494 (37.2)	477 (33.6)	217 (28.1)	102 (34.1)	
≥ 28 kg/m^2^	84 (9.8)	108 (10.6)	120 (9.0)	92 (6.5)	62 (8.0)	14 (4.7)	
Missing	37 (4.3)	52 (5.1)	73 (5.5)	82 (5.8)	35 (4.5)	10 (3.3)	
Mother’s education							< 0.0001
< 7 years	132 (15.3)	135 (13.2)	151 (11.4)	163 (11.5)	65 (8.4)	11 (3.7)	
7–12 years	530 (61.6)	606 (59.4)	804 (60.5)	835 (58.8)	464 (60.0)	191 (63.9)	
≥ 13 years	144 (16.7)	205 (20.1)	274 (20.6)	315 (22.2)	188 (24.3)	82 (27.4)	
Missing	55 (6.4)	75 (7.3)	99 (7.5)	107 (7.5)	56 (7.2)	15 (5.0)	
Father’s education							< 0.0001
< 7 years	80 (9.3)	69 (6.8)	101 (7.6)	80 (5.6)	36 (4.7)	7 (2.3)	
7–12 years	552 (64.1)	658 (64.4)	835 (62.9)	851 (59.9)	467 (60.4)	194 (64.9)	
≥ 13 years	180 (20.9)	223 (21.8)	297 (22.4)	385 (27.1)	214 (27.7)	85 (28.4)	
Missing	49 (5.7)	71 (7.0)	95 (7.2)	104 (7.3)	56 (7.2)	13 (4.3)	
Household income per month							< 0.0001
< 750 RMB	108 (12.5)	145 (14.2)	153 (11.5)	149 (10.5)	67 (8.7)	13 (4.3)	
751–1500 RMB	317 (36.8)	339 (33.2)	420 (31.6)	414 (29.2)	199 (25.7)	70 (23.4)	
1501–2500 RMB	217 (25.2)	246 (24.1)	336 (25.3)	335 (23.6)	204 (26.4)	91 (30.4)	
≥ 2501 RMB	150 (17.4)	208 (20.4)	307 (23.1)	400 (28.2)	243 (31.4)	110 (36.8)	
Missing	69 (8.0)	83 (8.1)	112 (8.4)	122 (8.6)	60 (7.8)	15 (5.0)	
Intervention							< 0.0001
No	288 (33.4)	440 (43.1)	695 (52.3)	729 (51.3)	361 (46.7)	116 (38.8)	
Yes	573 (66.6)	581 (56.9)	633 (47.7)	691 (48.7)	412 (53.3)	183 (61.2)	

BMI, body mass index; CMRS, cardiometabolic risk score; DBP, diastolic blood pressure; HDL-C, high-density lipoprotein cholesterol; LDL-C, low-density lipoprotein cholesterol; MAP, mean arterial pressure; SBP, systolic blood pressure; TC, total cholesterol; TG, triglyceride

*HDS was computed by summing sub-scores with each of the leading dietary predictors as one point according to their associations with CMRS. For example, more than the median intake of fruit was scored as 1 and equal or less as 0, if fruit intake was inversely associated with CMRS

^†^ANOVA was used to test the difference of continuous variables across healthy diet score and Chi-square for categorical variables

^‡^All such data were mean ± standard deviation

^§^All such data were frequency (percentage)

### Importance of contributors to CMRS

**Fig. 2:**
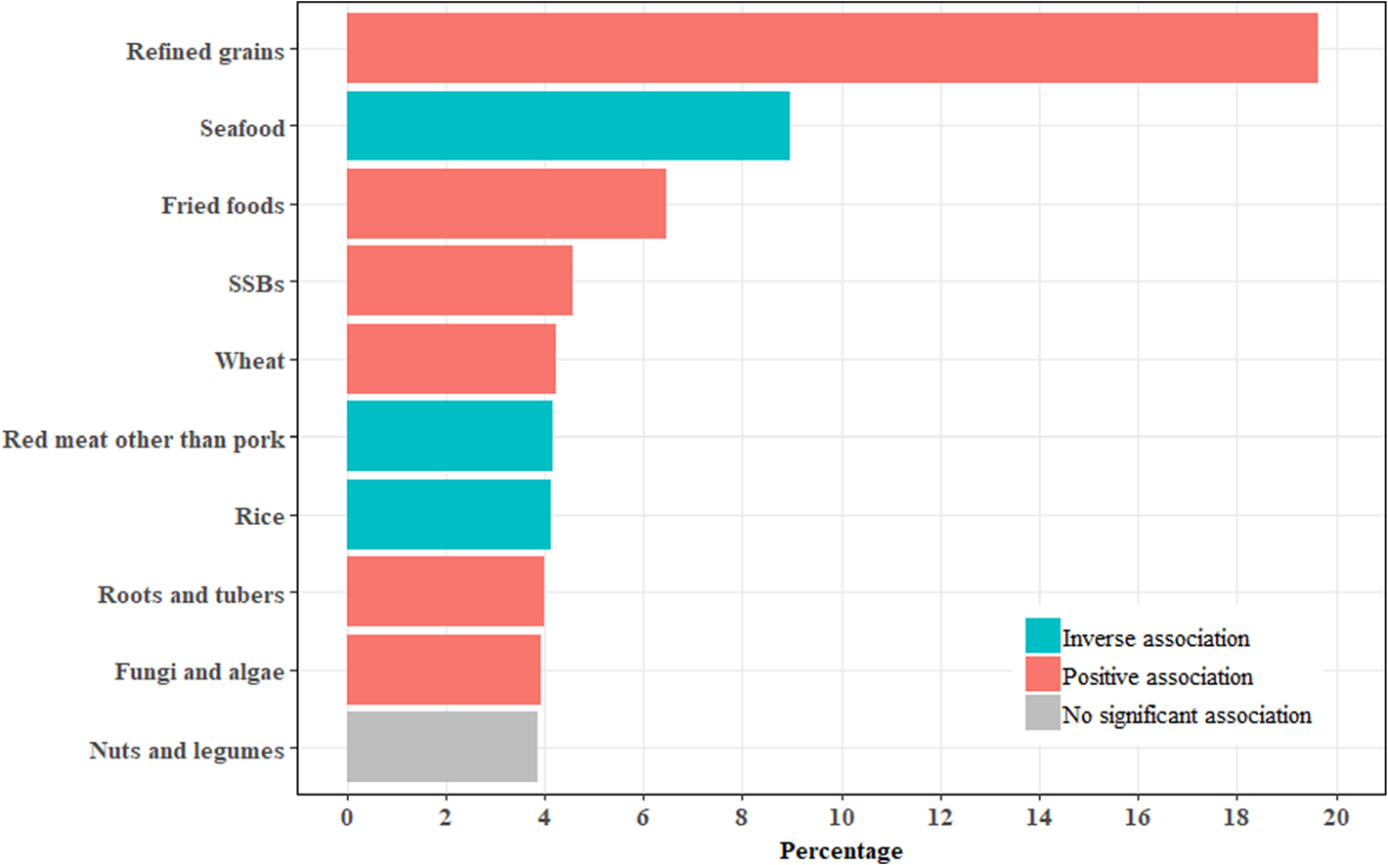
Leading dietary determinants for changes in cardiometabolic risk scores in children. This figure shows the contribution of the total variance in percentage by leading dietary determinants (selected from 26 food groups). Machine learning models including general linear regression model, random forest, and gradient boost machine were used to analyze the importance of dietary predictors for CMRS. Random forest had the highest prediction performance and this figure shows the leading dietary determinants derived from the random forest

Random Forest exhibited higher R-square compared with the other two machine learning models for CMRS (Table S4). Figure 2 depicts the leading predictors for CMRS as derived from Random Forest. The nine leading predictors for CMRS were refined grains, seafood, fried foods, SSBs, wheat, red meat other than pork, rice, fungi and algae, and roots and tubers with the contribution ranging from 3.9 to 19.6% of the total variance. These leading predictors were consistent with those identified by GBM and GLM (Table S5).

### Dietary intakes and CMRS and healthy diet score

Diets low in refined grains, fried foods, SSBs, wheat, fungi and algae, roots and tubers and high in seafood, rice, and red meat other than pork were associated with a favorable change in CMRS (Table 2). HDS was then computed by summing sub-scores with each of the nine leading healthy factors as one point (according to their associations with CMRS): refined grains (<median), seafood (>median), fried foods (<median), SSBs (<median), wheat (<median), red meat other than pork (>median), rice (>median), fungi and algae (<median), and roots and tubers (<median). HDS ranged from 0 to 9 with a higher level representing a healthier diet. HDS was also calculated by summing the weighted sub-scores according to the contribution derived from the Random Forest. The maximum sub-score of 1 was set at the levels of 0 (refined grains, fried foods, SSBs, wheat, fungi and algae, and roots and tubers) or above the 80th percentile (seafood, rice, and red meat other than pork) of the food intake. While the minimum score of 0 was set at the levels of above the 80th percentile (refined grains, fried foods, SSBs, wheat, fungi and algae, and roots and tubers) or 0 (seafood, rice, and red meat other than pork) of the food intake. Scores for the amounts between 0 and 1 were prorated linearly. The sub-scores were then weighted by multiplying the contribution (percentage of the total variance of the nine dietary predictors) of the corresponding dietary predictors (Table S6).

**Table 2:** Changes in cardiometabolic risk score during follow-up associated with dietary intakes at baseline

	Low intake (gram/100 kcal/day)	High intake (gram/100 kcal/day)	*P*-value*
Refined grains	0	> 0	
Participants	1453	3361	
CMRS^†^, Model 1^‡^	−0.24 ± 0.12^§^	− 0.03 ± 0.12	0.0024
CMRS, Model 2	− 0.12 ± 0.12	0.07 ± 0.11	0.0074
CMRS, Model 3	0.01 ± 0.14	0.21 ± 0.14	0.0058
Seafood	0	> 0	
Participants	2571	2243	
CMRS, Model 1	0.09 ± 0.11	− 0.37 ± 0.12	< 0.0001
CMRS, Model 2	0.22 ± 0.11	− 0.25 ± 0.11	< 0.0001
CMRS, Model 3	0.32 ± 0.14	− 0.14 ± 0.14	< 0.0001
Fried wheat/rice	0	> 0	
Participants	3892	922	
CMRS, Model 1	−0.22 ± 0.12	0.34 ± 0.13	< 0.0001
CMRS, Model 2	− 0.09 ± 0.11	0.34 ± 0.13	< 0.0001
CMRS, Model 3	0.04 ± 0.14	0.45 ± 0.15	< 0.0001
SSBs	0	> 0	
Participants	3472	1342	
CMRS, Model 1	−0.17 ± 0.12	0.07 ± 0.13	0.0008
CMRS, Model 2	−0.05 ± 0.11	0.18 ± 0.12	0.0007
CMRS, Model 3	0.08 ± 0.14	0.33 ± 0.15	0.0004
Wheat	≦4.66	> 4.66	
Participants	3048	1766	
CMRS, Model 1	−0.21 ± 0.12	0.07 ± 0.12	< 0.0001
CMRS, Model 2	− 0.05 ± 0.11	0.10 ± 0.12	0.0182
CMRS, Model 3	0.08 ± 0.14	0.22 ± 0.14	0.0433
Red meat other than pork	≦0.01	> 0.01	
Participants	3394	1420	
CMRS, Model 1	−0.03 ± 0.12	− 0.28 ± 0.12	0.0005
CMRS, Model 2	0.10 ± 0.11	− 0.19 ± 0.12	< 0.0001
CMRS, Model 3	0.23 ± 0.14	− 0.05 ± 0.14	< 0.0001
Rice	≦5.99	> 5.99	
Participants	1109	3705	
CMRS, Model 1	0.33 ± 0.13	− 0.25 ± 0.12	< 0.0001
CMRS, Model 2	0.46 ± 0.13	− 0.12 ± 0.11	< 0.0001
CMRS, Model 3	0.54 ± 0.15	− 0.01 ± 0.14	< 0.0001
Root and tuber	≦2.29	> 2.29	
Participants	2711	2103	
CMRS, Model 1	−0.14 ± 0.12	0.00 ± 0.13	0.0635
CMRS, Model 2	−0.00 ± 0.11	0.05 ± 0.12	0.41
CMRS, Model 3	0.14 ± 0.14	0.17 ± 0.14	0.63
Fungi and mushroom	0	> 0	
Participants	3244	1570	
CMRS, Model 1	−0.16 ± 0.12	0.03 ± 0.12	0.0058
CMRS, Model 2	−0.04 ± 0.11	0.12 ± 0.12	0.0195
CMRS, Model 3	0.10 ± 0.14	0.25 ± 0.14	0.0212
Nuts and legumes	0	> 0	
Participants	1946	2868	
CMRS, Model 1	−0.16 ± 0.12	− 0.07 ± 0.12	0.17
CMRS, Model 2	− 0.00 ± 0.11	0.02 ± 0.11	0.79
CMRS, Model 3	0.13 ± 0.14	0.15 ± 0.14	0.78

^*^The change in CMRS was calculated by subtracting the result at baseline from that at follow-up

^†^GLM was used to estimate multivariable-adjusted means and standard errors of cardiometabolic risk factors between quintiles. Benjamin-Hochberg’s procedure was used to control the false discovery rate at level 5% for multiple comparisons with the P-value cut-off point of significance was 0.0233 for change in CMRS (Model 3)

^‡^Model 1 was adjusted for classes in school as clustering effects and characteristics of individuals including age, sex, and corresponding CMR factor at baseline as fixed effects; Model 2 was adjusted for Model 1 plus puberty, grade, intervention, BMI, physical activity, and energy intake at baseline as fixed effects; Model 3 was adjusted for Model 2 plus birthweight, household income, mother’s education, father’s education, mother’s BMI, and father’s BMI as fixed effects

^§^All these data are means ± standard errors of change in CMRS

### Healthy diet score and CMR factors

High HDS at baseline was associated with favorable changes in CMRS, BMI, PBF, SBP, DBP, MAP, HDL-C, fasting glucose, insulin, and HOMA-IR. There was a positive association between HDS at baseline and changes in TC and LDL-C (Table 3). High weighted HDS at baseline was associated with favorable changes in CMRS, BMI, PBF, SBP, DBP, MAP, HDL-C, fasting glucose, insulin, and HOMA-IR (Table S7). Improved HDS was associated with favorable changes in BMI, SBP, DBP, MAP, fasting glucose, insulin, HOMA-IR, and CMRS (Table S8).

**Table 3:** Changes in cardiometabolic risk factors during follow-up associated with Healthy Diet Score at baseline

	Healthy Diet Score						*P*-trend*
	≤3	4	5	6	7	≥8	
Change in BMI^†^							
Participants	842	1011	1307	1381	766	298	
β (95% CI), Model 1^‡^	0	−0.03 (− 0.08, 0.03)^§^	− 0.06 (− 0.11, − 0.00)	− 0.07 (− 0.12, − 0.01)	−0.08 (− 0.14, − 0.02)	−0.09 (− 0.17, − 0.02)	0.0004
β (95% CI), Model 2	0	− 0.02 (− 0.08, 0.04)	−0.06 (− 0.11, − 0.00)	−0.07 (− 0.13, − 0.01)	−0.07 (− 0.13, − 0.01)	−0.09 (− 0.17, − 0.02)	0.0007
β (95% CI), Model 3	0	− 0.02 (− 0.08, 0.04)	− 0.06 (− 0.11, − 0.00)	−0.06 (− 0.12, − 0.01)	−0.07 (− 0.13, − 0.01)	− 0.08 (− 0.16, − 0.00)	0.0041
Change in WC							
Participants	844	1004	1304	1374	764	299	
β (95% CI), Model 1	0	−0.06 (− 0.11, − 0.01)	−0.01 (− 0.06, 0.03)	− 0.04 (− 0.08, 0.01)	−0.04 (− 0.09, 0.01)	−0.05 (− 0.12, 0.01)	0.18
β (95% CI), Model 2	0	−0.05 (− 0.10, − 0.01)	−0.01 (− 0.06, 0.03)	−0.04 (− 0.09, 0.00)	− 0.03 (− 0.08, 0.01)	−0.04 (− 0.10, 0.02)	0.23
β (95% CI), Model 3	0	−0.05 (− 0.10, − 0.01)	−0.02 (− 0.06, 0.03)	−0.04 (− 0.09, 0.00)	− 0.04 (− 0.08, 0.01)	− 0.05 (− 0.11, 0.01)	0.15
Change in PBF							
Participants	813	977	1275	1349	743	294	
β (95% CI), Model 1	0	0.01 (− 0.06, 0.08)	0.00 (− 0.07, 0.07)	− 0.08 (− 0.15, − 0.01)	−0.12 (− 0.19, − 0.04)^bc^	−0.08 (− 0.18, 0.01)	< 0.0001
β (95% CI), Model 2	0	−0.00 (− 0.07, 0.07)	−0.01 (− 0.08, 0.06)	− 0.10 (− 0.17, − 0.03)	−0.14 (− 0.21, − 0.06)^abc^	− 0.10 (− 0.19, − 0.00)	< 0.0001
β (95% CI), Model 3	0	0.00 (− 0.07, 0.07)	−0.01 (− 0.08, 0.06)	−0.10 (− 0.17, − 0.03)	− 0.13 (− 0.21, − 0.06)^bc^	− 0.09 (− 0.18, 0.01)	< 0.0001
Change in SBP							
Participants	847	1010	1307	1369	760	298	
β (95% CI), Model 1	0	−0.08 (− 0.18, 0.01)	− 0.18 (− 0.27, − 0.09)^a^	−0.33 (− 0.42, − 0.24)^abc^	−0.42 (− 0.52, − 0.32)^abc^	−0.53 (− 0.66, − 0.41)^abcd^	< 0.0001
β (95% CI), Model 2	0	− 0.07 (− 0.17, 0.02)	−0.17 (− 0.25, − 0.08)^a^	−0.29 (− 0.38, − 0.21)^ab^	−0.39 (− 0.48, − 0.29)^abc^	−0.48 (− 0.60, − 0.36)^abc^	< 0.0001
β (95% CI), Model 3	0	− 0.07 (− 0.16, 0.02)	−0.16 (− 0.25, − 0.07)	−0.28 (− 0.37, − 0.19)^ab^	−0.37 (− 0.46, − 0.27)^abc^	−0.46 (− 0.58, − 0.34)^abc^	< 0.0001
Change in DBP							
Participants	848	1010	1309	1373	760	298	
β (95% CI), Model 1	0	−0.03 (−0.12, 0.07)	−0.17 (− 0.26, − 0.08)^a^	−0.36 (− 0.44, − 0.27)^abc^	−0.45 (− 0.54, − 0.35)^abc^	−0.49 (− 0.61, − 0.37)^abc^	< 0.0001
β (95% CI), Model 2	0	− 0.03 (− 0.12, 0.07)	−0.16 (− 0.25, − 0.07)	−0.32 (− 0.41, − 0.23)^abc^	−0.43 (− 0.53, − 0.33)^abc^	−0.46 (− 0.59, − 0.34)^abc^	< 0.0001
β (95% CI), Model 3	0	−0.03 (− 0.12, 0.07)	−0.16 (− 0.25, − 0.07)	−0.32 (− 0.41, − 0.23)^abc^	−0.43 (− 0.52, − 0.33)^abc^	−0.46 (− 0.58, − 0.34)^abc^	< 0.0001
Change in MAP							
Participants	847	1010	1307	1371	759	298	
β (95% CI), Model 1	0	−0.05 (−0.14, 0.05)	−0.19 (− 0.28, − 0.10)^a^	−0.37 (− 0.46, − 0.28)^abc^	−0.47 (− 0.56, − 0.37)^abc^	−0.55 (− 0.67, − 0.42)^abc^	< 0.0001
β (95% CI), Model 2	0	− 0.04 (− 0.14, 0.05)	−0.18 (− 0.26, − 0.09)^a^	−0.34 (− 0.42, − 0.25)^abc^	−0.44 (− 0.54, − 0.35)^abc^	−0.51 (− 0.63, − 0.38)^abc^	< 0.0001
β (95% CI), Model 3	0	−0.04 (− 0.14, 0.05)	−0.17 (− 0.26, − 0.09)^a^	−0.33 (− 0.42, − 0.24)^abc^	−0.43 (− 0.53, − 0.34)^abc^	−0.50 (− 0.62, − 0.38)^abc^	< 0.0001
Change in TC							
Participants	798	945	1227	1306	719	278	
β (95% CI), Model 1	0	−0.01 (−0.08, 0.07)	0.05 (−0.01, 0.12)	0.19 (0.13, 0.26)^abc^	0.20 (0.13, 0.28)^abc^	0.21 (0.12, 0.31)^abc^	< 0.0001
β (95% CI), Model 2	0	−0.01 (− 0.09, 0.06)	0.03 (− 0.04, 0.10)	0.17 (0.10, 0.24)^abc^	0.18 (0.11, 0.26)^abc^	0.20 (0.11, 0.30)^ab^	< 0.0001
β (95% CI), Model 3	0	−0.01 (− 0.08, 0.06)	0.03 (− 0.04, 0.10)	0.17 (0.10, 0.23)^abc^	0.17 (0.09, 0.24)^abc^	0.18 (0.08, 0.27)^b^	< 0.0001
Change in HDL-C							
Participants	798	943	1223	1309	720	278	
β (95% CI), Model 1	0	0.02 (−0.08, 0.12)	0.05 (− 0.05, 0.15)	0.28 (0.18, 0.37)^abc^	0.42 (0.31, 0.53)^abc^	0.37 (0.23, 0.50)^abc^	< 0.0001
β (95% CI), Model 2	0	0.01 (−0.10, 0.11)	0.03 (−0.07, 0.12)	0.21 (0.11, 0.31)^abc^	0.36 (0.26, 0.47)^abc^	0.30 (0.17, 0.44)^abc^	< 0.0001
β (95% CI), Model 3	0	0.01 (−0.09, 0.11)	0.02 (−0.07, 0.12)	0.20 (0.11, 0.30)^bc^	0.36 (0.25, 0.46)^abc^	0.28 (0.15, 0.42)^abc^	< 0.0001
Change in LDL-C							
Participants	797	945	1229	1307	718	279	
β (95% CI), Model 1	0	0.02 (−0.05, 0.10)	0.12 (0.05, 0.20)	0.30 (0.22, 0.37)^abc^	0.42 (0.34, 0.50)^abc^	0.47 (0.37, 0.58)^abcd^	< 0.0001
β (95% CI), Model 2	0	0.03 (−0.05, 0.11)	0.12 (0.05, 0.20)	0.32 (0.24, 0.39)^abc^	0.42 (0.34, 0.50)^abc^	0.49 (0.38, 0.59)^abc^	< 0.0001
β (95% CI), Model 3	0	0.03 (−0.05, 0.11)	0.11 (0.04, 0.19)	0.30 (0.22, 0.37)^abc^	0.39 (0.31, 0.47)^abc^	0.44 (0.34, 0.55)^abc^	< 0.0001
Change in TG							
Participants	798	945	1228	1308	720	279	
β (95% CI), Model 1	0	−0.02 (−0.12, 0.07)	0.00 (−0.08, 0.09)	− 0.06 (− 0.15, 0.03)	−0.12 (− 0.22, − 0.03)	−0.04 (− 0.17, 0.08)	0.50
β (95% CI), Model 2	0	−0.02 (− 0.11, 0.07)	0.01 (− 0.08, 0.09)	−0.03 (− 0.11, 0.06)	−0.10 (− 0.19, − 0.00)	0.00 (− 0.12, 0.12)	0.96
β (95% CI), Model 3	0	−0.02 (− 0.11, 0.07)	0.01 (− 0.08, 0.09)	−0.03 (− 0.12, 0.06)	−0.11 (− 0.20, − 0.01)	−0.01 (− 0.13, 0.11)	0.49
Change in fasting glucose							
Participants	796	943	1229	1311	720	278	
β (95% CI), Model 1	0	−0.10 (−0.18, − 0.02)	−0.18 (− 0.26, − 0.11)^a^	−0.25 (− 0.32, − 0.17)^ab^	−0.23 (− 0.31, − 0.15)^a^	−0.22 (− 0.33, − 0.12)^a^	< 0.0001
β (95% CI), Model 2	0	−0.09 (− 0.17, − 0.01)	−0.16 (− 0.23, − 0.08)^a^	−0.21 (− 0.29, − 0.14)^ab^	−0.21 (− 0.29, − 0.13)^ab^	−0.21 (− 0.32, − 0.11)^a^	< 0.0001
β (95% CI), Model 3	0	−0.09 (− 0.16, − 0.01)	−0.16 (− 0.23, − 0.08)^a^	−0.21 (− 0.29, − 0.14)^ab^	−0.21 (− 0.29, − 0.13)^ab^	−0.22 (− 0.32, − 0.11)^a^	< 0.0001
Change in insulin							
Participants	700	810	1062	1148	667	257	
β (95% CI), Model 1	0	−0.08 (−0.24, 0.07)	−0.13 (− 0.28, 0.01)	−0.35 (− 0.49, − 0.21)^abc^	−0.56 (− 0.72, − 0.40)^abc^	−0.64 (− 0.84, − 0.44)^abc^	< 0.0001
β (95% CI), Model 2	0	− 0.07 (− 0.22, 0.08)	−0.09 (− 0.23, 0.05)	−0.28 (− 0.42, − 0.13)	−0.48 (− 0.63, − 0.33)^abc^	−0.55 (− 0.74, − 0.36)^abc^	< 0.0001
β (95% CI), Model 3	0	− 0.06 (− 0.20, 0.09)	−0.07 (− 0.21, 0.07)	−0.25 (− 0.39, − 0.11)	−0.45 (− 0.60, − 0.30)^abc^	−0.52 (− 0.71, − 0.32)^abc^	< 0.0001
Change in HOMA-IR							
Participants	699	810	1062	1148	667	256	
β (95% CI), Model 1	0	−0.10 (−0.25, 0.04)	−0.17 (− 0.31, − 0.03)	−0.39 (− 0.53, − 0.25)^abc^	−0.59 (− 0.74, − 0.44)^abc^	−0.67 (− 0.86, − 0.48)^abc^	< 0.0001
β (95% CI), Model 2	0	− 0.09 (− 0.24, 0.05)	−0.13 (− 0.27, 0.01)	−0.32 (− 0.46, − 0.18)^a^	−0.52 (− 0.67, − 0.37)^abc^	−0.58 (− 0.77, − 0.39)^abc^	< 0.0001
β (95% CI), Model 3	0	− 0.08 (− 0.22, 0.07)	−0.11 (− 0.25, 0.02)	−0.30 (− 0.43, − 0.16)^a^	−0.49 (− 0.63, − 0.34)^abc^	−0.55 (− 0.73, − 0.36)^abc^	< 0.0001
Change in CMRS							
Participants	713	852	1118	1198	668	265	
β (95% CI), Model 1	0	−0.25 (−0.48, − 0.01)	−0.38 (− 0.61, − 0.16)	−0.94 (− 1.16, − 0.72)^abc^	−1.22 (− 1.46, − 0.97)^abc^	−1.21 (− 1.51, − 0.90)^abc^	< 0.0001
β (95% CI), Model 2	0	−0.21 (− 0.44, 0.01)	−0.32 (− 0.53, − 0.11)	−0.79 (− 1.00, − 0.58)^abc^	− 1.10 (− 1.33, − 0.87)^abc^	−1.05 (− 1.34, − 0.76)^abc^	< 0.0001
β (95% CI), Model 3	0	−0.22 (− 0.44, 0.01)	−0.31 (− 0.52, − 0.10)	−0.77 (− 0.98, − 0.55)^abc^	− 1.08 (− 1.31, − 0.85)^abc^	−1.02 (− 1.31, − 0.73)^abc^	< 0.0001

BMI, body mass index; CMRS, cardiometabolic risk score; DBP, diastolic blood pressure; HOMA-IR, homeostatic model assessment of insulin resistance; HDL-C, high-density lipoprotein cholesterol; LDL-C, low-density lipoprotein cholesterol; MAP, mean arterial pressure; SBP, systolic blood pressure; SE, standard error; TC, total cholesterol; TG, triglyceride

*GLM was used to estimate beta coefficients (β) and 95% CIs of cardiometabolic risk factors between quintiles. Benjamin-Hochberg’s procedure was used to control the false discovery rate at level 5% for multiple comparisons with the *P*-value cut-off point of significance was 0.0433 for HDS and changes in CMR factors (Model 3)

^†^Changes in CMR factors were calculated by subtracting the results at baseline from those at follow-up

^‡^Model 1 was adjusted for classes in school as clustering effects and characteristics of individuals including age, sex, and corresponding CMR factor at baseline as fixed effects; Model 2 was adjusted for Model 1 plus puberty, grade, intervention, BMI, physical activity, and intake of energy, fiber, vegetable, fruit, pork, legumes, and nuts at baseline as fixed effects; Model 3 was adjusted for Model 2 plus birthweight, household income, mother’s education, father’s education, mother’s BMI, and father’s BMI as fixed effects

^§^All these data are β (95% CI) of changes in CMR factors

^abcd^Bonferroni Post-hoc test was used to examine the difference between every two groups of the healthy diet score with ^a^ indicating significance compared with HDS ≤ 3, ^b^ indicating significance compared with HDS = 4, ^c^ indicating significance compared with HDS = 5, and ^d^ indicating significance compared with HDS = 6. The comparisons with HDS = 7 were also conducted, but no significant associations were found

### Moderation analysis

**Fig. 3:**
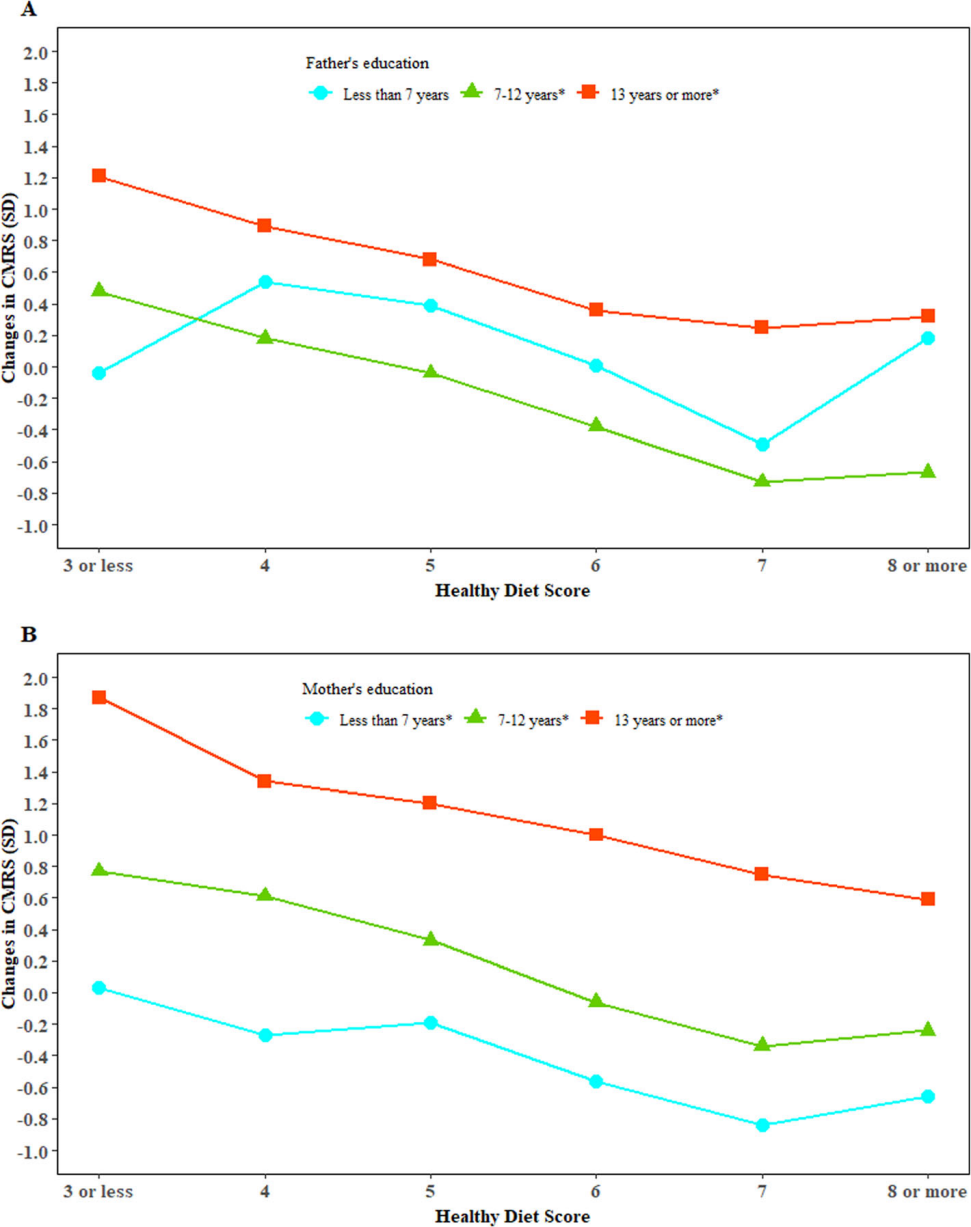
Associations between healthy diet score and changes in cardiometabolic risk score modified by parental education. CMRS, cardiometabolic risk score; SD, standard deviation. The general linear regression model was used to test the interaction adjusted for classes in schools as random effects and characteristics of the individuals including age, sex, intervention, grade, puberty, BMI, physical activity, CMRS, and intake of energy, fiber, vegetable, fruit, pork, legumes, and nuts at baseline, birth weight, breastfeeding, household income, or parental BMI and education. We examined whether the association between healthy diet score and CMRS was modified by sex, grade, birthweight, household income, parental BMI, and parental education and a significant interaction were observed only for healthy diet score and parental education. *represents there is a significant association between healthy diet score and change in CMRS

The inverse association between HDS and CMRS was stronger in children whose parents had higher education (Fig. 3). No significant interaction between HDS and sex, grade, birthweight, household income, or parental BMI for change in CMRS was observed.

### Sensitivity analysis

High HDS at baseline was associated with favorable changes in CMRS, PBF, SBP, DBP, MAP, HDL-C, TG, insulin, and HOMA-IR in the control group (Table S9).

### External validation

We examined the association of HDS with available CMR factors among 4530 children aged 6–13 years from CHNS. Baseline HDS was inversely associated with BMI, WC, SBP, DBP, and MAP at baseline and the change in DBP (Table S10).

## Discussion

In this longitudinal analysis of children with large sample size, we found the nine leading healthy dietary determinants for CMRS were diets low in refined grains, fried foods, SSBs, wheat, fungi and algae, and roots and tubers, and high in seafood, rice, and red meat other than pork. We created an HDS based on these leading determinants that were shown to be a strong predictor for changes in 10 out of 14 CMR factors examined. The inverse association between HDS and CMRS was more likely to be evident in children whose parents had high education. The predictive ability of our HDS on several CMRS factors was validated in children from CHNS.

Previous studies have shown that diets high in glycemic index are associated with high CMR in children [29]. Our findings agree with these studies showing that high consumption of refined grains, fried foods, SSBs, roots and tubers, or wheat was associated with a higher increase in CMRS. The positive association between SSBs intake and CMR factors has been reported in many studies [16, 17, 30]. Although refined grains and wheat were not linked to CMR factors in children, their harmful effect on CMR has been reported in adults [31]. Foods being fried have lower nutrients and higher energy density than those being boiled or steamed [32, 33]. Several cohort studies in adults showed that higher consumption of fried foods was associated with an increased risk of obesity, type 2 diabetes, and cardiovascular diseases [34, 35]. Our study supports the dietary guidelines that diets low in glycemic index are beneficial for the prevention of CMR factors.

Seafood, rice, and red meat other than pork are major sources of protein that plays an important role in child growth and development. High consumption of fish has been recommended for the prevention of CMR factors in adults because fish is rich in protein, omega-3 fatty acids, and minerals [12, 13]. Our further analysis shows that red meat other than pork intake was only significantly associated with two (pork and milk) out of 25 food groups (Table S11). The intake of pork and milk was not a significant predictor of CMRS suggesting that the red meat other than pork intake was independently associated with CMRS. Processed but not unprocessed red meat is associated with an increased risk of obesity and related CMR in previous studies suggesting that unprocessed red meat other than pork may be considered as part of a healthy balanced diet in children considering its high contents in protein [36]. Compared with the harmful effect of high wheat intake, high rice (white and brown) intake resulted in a beneficial change in CMRS in our study, which may be partly due to the difference in nutrient composition between rice and wheat [21]. The divergent associations of rice and wheat intake with CMRS may also be attributable to the fact that they are associated with different dietary patterns. For example, we found high rice intake was associated with a higher intake of vegetables, fish, and poultry and a lower intake of fried foods, beverages, refined grains, and edible fungus and algae (Table S12). In contrast, high wheat intake was associated with a higher intake of fried foods, refined grains, roots and tubers, and a lower intake of fish, pork, milk, and vegetables (Table S13). Our findings highlight the importance of high consumption of seafood, rice, and red meat other than pork on the prevention of CMR factors in children.

The association between mushroom consumption and CMR factors is inconsistent between studies with the largest longitudinal study showing no significant association [37, 38]. The association of edible fungus and algae with CMR is less known. The positive association of fungi and algae with CMR factors in our study may be partly attributed to the harmful constituents in some of them [39]. However, more research needs to warrant our findings.

Dietary patterns derived by posterior methods including principal component analysis, cluster analysis, and latent class analysis have been linked to CMR factors in children [15–17, 40]. These studies showed that Western dietary pattern, high energy-dense pattern, or sweet dietary pattern were associated with high CMR [15–17], whereas vegetable and the wholemeal pattern was associated with favorable changes in CMR factors [40]. Although these findings may imply which food groups are associated with CMR factors, these dietary patterns can hardly be obtained in other studies. In contrast, priori patterns based on dietary guidelines may be applied to different studies and the findings are comparable [18]. An inverse association between adherence to Dietary Approaches to Stop Hypertension (DASH) Dietary Pattern or Mediterranean pattern and CMR has been observed in some studies [41, 42], but not in other studies [18, 43]. Therefore, establishing an efficient HDS based on evidence to predict CMR factors in children is urgent. Our HDS created based on the leading determinants of CMRS was strongly associated with 10 out of 14 CMR factors. Although no significant association of baseline HDS with baseline CMRS was observed, both high baseline HDS and improved HDS were associated with favorable changes in most CMR factors in our study. Furthermore, validation analysis in children from CHNS showed that higher baseline HDS was associated with lower BMI, WC, SBP, DBP, and MAP in the cross-sectional analysis and a lower increase in DBP only in the longitudinal analysis. The weak association between HDS and CMR factors in the longitudinal analysis might be due to the small variation of HDS and small available sample size in some HDS subgroups. We found higher parental education and higher HDS resulted in more decrease in CMRS suggesting the importance of the involvement of parents with high education and children whose parents with low education are more needed in care.

The strengths of the present study included the large sample size and the measurement of multiple CMR factors and dietary intakes assessed at both baseline and follow-up. To our knowledge, this is the first study to identify leading dietary determinants of CMRS in children using machine learning techniques. We also created an HDS based on medians of leading determinants that were strongly predictive of most CMR factors. This score was also validated in children from CHNS. The study has several limitations. Firstly, 24 h of food records are limited by not accounting for seasonal variation of dietary intakes especially fruits and vegetables. However, the dietary intakes are comparable between individuals given all data were collected in May of the year. Furthermore, our HDS was validated in a Chinese population, but whether HDS was predictive of CMR needs to be examined in other ethnic groups. The validation dataset is also limited by only having several CMR factors measured at baseline and follow-up, which makes it impossible to compute CMRS. Thirdly, several food items such as algae and fungi were not frequently consumed by people in countries other than Asia therefore the HDS was not applied to these populations. However, nuts, milk, and vegetables (11th, 12th, and 13th leading predictors in our study) instead of these food items may be included in the calculation of the HDS. Fourthly, the importance of an individual food for the CMRS is deriving partly from that food but also from other foods it is correlated with, which was not accounted for in our machine learning analysis. Fithly, the follow-up period of our study (one year) is relatively short to judge the effect of dietary factors on change in CMR, therefore longitudinal studies with long-term follow-up are needed to warrant our findings. Finally, because of the observational nature of the analysis in the present study, causal relations could not be established based on our findings.

## Conclusions

Diets high in seafood, rice, and red meat other than pork and low in refined grains, fried foods, SSBs, wheat, fungi and algae, roots and tubers are leading healthy diet factors for changes in CMR factors in children. HDS based on these leading dietary determinants is strongly predictive of CMR factors.

## Supplementary information

**Additional file 1.**

## Data Availability

The datasets generated and/or analyzed during the current study are not publicly available due to the privacy protection of the participants but are available from the corresponding author on reasonable request.

## Abbreviations

BMI: body mass index
CMRS: cardiometabolic risk score
DBP: diastolic blood pressure
HDL-C: high-density lipoprotein cholesterol
HDS: healthy diet score
LDL-C: low-density lipoprotein cholesterol
MAP: mean arterial pressure
SBP: systolic blood pressure
SEM: standard error of the mean
SSBs: sugar-sweetened beverages
TC: total cholesterol
TG: triglyceride
