# Supplementary material for: Leading dietary determinants identified using machine learning techniques and a healthy diet score for changes in cardiometabolic risk factors in children: a longitudinal analysis
Source: Nutr J. 2020 Sep 19;19:105. doi: 10.1186/s12937-020-00611-2 (PMC7502204; doi:10.1186/s12937-020-00611-2)
Supplement: Supplementary file 1 — Additional file 1. [file 12937_2020_611_MOESM1_ESM.docx]

**Healthy diet score based on leading dietary determinants identified using machine learning techniques for changes in cardiometabolic risk factors in children: a longitudinal analysis**

**Nutrition Journal**

**Author Names:** Xianwen Shang^1-3^, Yanping Li^4^, Haiquan Xu^5^, Qian Zhang^1^, Ailing Liu^1^, Songming Du^6^, Hongwei Guo^7^, Guansheng Ma^8^

**Author Affiliations:**

^1^National Institute for Nutrition and Health, Chinese Center for Disease Control and Prevention, Beijing, China.

^2^School of Behavioural and Health Sciences, Australian Catholic University, Australia

^3^Department of Medicine (Royal Melbourne Hospital), University of Melbourne, Australia

^4^Department of Nutrition, Harvard T. H. Chan School of Public Health, Boston, MA, USA.

^5^Institute of food and nutrition development, Ministry of Agriculture and Rural Affairs, Beijing, China.

^6^Chinese Nutrition Society, Beijing, China.

^7^School of Public Health, Fudan University, Shanghai, China.

^8^Department of Nutrition and Food Hygiene, School of Public Health, Peking University, Beijing, China.

**Correspondence Author:** Professor Guansheng Ma, Department of Nutrition and Food Hygiene, School of Public Health, Peking University, 38 Xue Yuan Road, Beijing, 100191, China. Tel: +86-10-82805266; Fax: +86-10-82801620. Email: [mags@bjmu.edu.cn](mailto:mags@bjmu.edu.cn).

**Supplementary materials**

**Table S1. Combinations of the Hyper-parameters with Best Performance for Machine Learning Methods**

**Table S2. P values for interaction between sex and healthy dietary score for changes in cardiometabolic risk factors**

**Table S3. P values for interaction between intervention and healthy dietary score for changes in cardiometabolic risk factors**

**Table S3. P values for interaction between intervention and healthy dietary score for changes in cardiometabolic risk factors**

**Table S4. R-square and RMSE by Different Machine Learning Methods**

**Table S5. Leading dietary determinants for changes in the cardiometabolic risk score identified using GBM and linear regression model**

**Table S6. Median, range, and quantile intake of leading dietary determinants**

**Table S7. Changes in cardiometabolic risk factors during follow-up associated with weighted Healthy Diet Score at baseline**

**Table S8. Changes in cardiometabolic risk factors associated with change in healthy dietary score**

**Table S9. Changes in cardiometabolic risk factors associated with healthy dietary score at baseline in the control group**

**Table S10. Validation of healthy dietary score in children from China Nutrition and Health Survey**

**Table S11. Red meat other than pork and other food items**

**Table S12. Rice and other food items**

**Table S13. Wheat and other food items**

**Table S1. Combinations of the Hyper-parameters with Best Performance for Machine Learning Methods^*^**

|  | **GLM** | **Random forest** | **GBM** |
| --- | --- | --- | --- |
| Combination of parameters | nfolds=5, alpha=0.2, lambda=0.1 | max_depth=6, mtries=9, seed = 1, nfolds=5, ntree = 500 | ntrees = 500, nfolds = 5, seed = 1, learn_rate=0.08, max_depth=2, sample_rate=0.5, col_sample_rate=0.6 |

^*^These combinations of the hyper-parameters with best performance would then be separately applied in the machine methods in the final analysis.

**Table S2. P values for interaction between sex and healthy dietary score for changes in cardiometabolic risk factors***

| Changes in CMR factors | Model 1 | Model 2 | Model 3 |
| --- | --- | --- | --- |
| BMI | 0.54 | 0.54 | 0.63 |
| WC | 0.30 | 0.43 | 0.61 |
| PBF | 0.59 | 0.68 | 0.63 |
| SBP | 0.23 | 0.16 | 0.17 |
| DBP | 0.61 | 0.32 | 0.39 |
| MAP | 0.73 | 0.44 | 0.51 |
| TC | 0.62 | 0.69 | 0.70 |
| HDL-C | 0.26 | 0.57 | 0.57 |
| LDL-C | 0.16 | 0.20 | 0.18 |
| Log TG | 0.80 | 0.92 | 0.90 |
| Fasting glucose | 0.11 | 0.09 | 0.10 |
| Log insulin | 0.17 | 0.42 | 0.45 |
| Log HOMA-IR | 0.15 | 0.35 | 0.38 |
| CMRS | 0.67 | 0.82 | 0.88 |

BMI, body mass index; CMRS, cardiometabolic risk score; DBP, diastolic blood pressure; HOMA-IR, homeostatic model assessment of insulin resistance; HDL-C, high-density lipoprotein cholesterol; LDL-C, low-density lipoprotein cholesterol; MAP, mean arterial pressure; SBP, systolic blood pressure; TC, total cholesterol; TG, triglyceride.

*GLM was used to test the interaction between sex and healthy dietary score for changes in cardiometabolic risk factors. Model 1 was adjusted for classes in school as clustering effects and characteristics of individuals including age, sex, and corresponding CMR factor at baseline as fixed effects. Model 2 was adjusted for Model 1 plus puberty, grade, intervention, BMI, physical activity, and intake of energy, fiber, vegetable, fruit, pork, legumes, and nuts at baseline as fixed effects. Model 3 was adjusted for Model 2 plus birthweight, household income, mother’s education, father’s education, mother’s BMI, and father’s BMI as fixed effects.

**Table S3. P values for interaction between intervention and healthy dietary score for changes in cardiometabolic risk factors***

| Changes in CMR factors | Model 1^†^ | Model 2^†^ | Model 3^†^ |
| --- | --- | --- | --- |
| BMI | 0.10 | 0.17 | 0.17 |
| WC | 0.06 | 0.14 | 0.16 |
| PBF | 0.02 | 0.03 | 0.03 |
| SBP | 0.37 | 0.21 | 0.18 |
| DBP | 0.22 | 0.19 | 0.16 |
| MAP | 0.16 | 0.12 | 0.10 |
| TC | 0.06 | 0.11 | 0.15 |
| HDL-C | 0.02 | 0.03 | 0.04 |
| LDL-C | **<0.0001** | **<0.0001** | **<0.0001** |
| Log TG | 0.07 | 0.10 | 0.10 |
| Fasting glucose | **<0.0001** | **<0.0001** | **<0.0001** |
| Log insulin | 0.66 | 0.60 | 0.65 |
| Log HOMA-IR | 0.69 | 0.65 | 0.70 |
| CMRS | 0.08 | 0.05 | 0.06 |

*GLM was used to test the interaction between intervention and healthy dietary score for changes in cardiometabolic risk factors adjusted for classes in schools as clustering effects and characteristics of the individuals including age, sex, grade, corresponding CMR factor at baseline, physical activity, total energy intake, birthweight, household income, mother’s education, father’s education, mother’s BMI, and father’s BMI as fixed effects.

^†^We used Benjamin-Hochberg procedure was used to control the false discovery rate at level 5% for multiple comparisons with the P value cut-off point of significance was 0.01 all three models.

**Table S4. R-square and RMSE by Different Machine Learning Methods^*^**

|  | RMSE**^†^** | R-square (%)**^‡^** | MAE**^¶^** |
| --- | --- | --- | --- |
| Random forest | 2.579 | 7.7 | 2.042 |
| Gradient boosting machine | 2.579 | 7.6 | 2.037 |
| GLM | 2.605 | 5.8 | 2.070 |

CMRS, cardiometabolic risk score; MAE, mean absolute error; RMSE, root mean square error

^*^50% of all participants were randomly selected as training data and the remaining as testing data.

**^†^**Low RMSE indicates high prediction ability.

**^‡^**High R-squared indicates high prediction ability. R-squared (%) stands for the percentage of total variance explained by involved predictors in models and we used R-squared to rank the performance of machine learning models.

**^¶^**Low MAE indicates high prediction ability.

**Table S5. Leading dietary determinants for changes in the cardiometabolic risk score identified using gradient boost machine and linear regression model***

|  |  | GBM |  | GLM |  |
| --- | --- | --- | --- | --- | --- |
| Ranking | Predictor | Variance  explained (%) |  | Predictor | Variance  explained (%) |
| 1 | Refined grains | 14 |  | Refined grains | 25.1 |
| 2 | Beverages | 6.7 |  | Seafood | 12.7 |
| 3 | Seafood | 6.5 |  | Fried foods | 8.5 |
| 4 | Fried foods | 5.2 |  | Rice | 6.1 |
| 5 | Eggs | 4.4 |  | Red meat other than pork | 6.1 |
| 6 | Wheat | 4.3 |  | Fungi and algae | 5.6 |
| 7 | Other cereals | 4.3 |  | Wheat | 5.4 |
| 8 | Fungi and algae | 4.1 |  | Yogurt | 5.0 |
| 9 | Roots and tubers | 4.0 |  | Beverages | 4.5 |
| 10 | Candy and sugar | 3.9 |  | Offal | 3.9 |

*This table shows the contribution of the total variance in percentage by top 10 leading dietary determinants (selected from 26 food groups). Machine learning models including general linear regression model and gradient boost machine to analyze the importance of dietary predictors for CMRS.

**Table S6. Median, range, and quantile intake of leading dietary determinants**

| Food groups | Median | Range | Interquartile range | 80^th^ percentile |
| --- | --- | --- | --- | --- |
| Refined grains (gram/100 kcal/day) | 0 | 0-54.78 | 0-2.73 | 4.19 |
| Fish (gram/100 kcal/day) | 0.01 | 0-47.67 | 0-3.27 | 4.20 |
| Fried foods (gram/100 kcal/day) | 0 | 0-19.05 | 0, 0 | 0.64 |
| Sugar-sweetened beverages (gram/100 kcal/day) | 0 | 0-67.72 | 0-1.60 | 2.57 |
| Rice (gram/100 kcal/day) | 5.99 | 0-107.63 | 3.18-10.42 | 11.73 |
| Wheat (gram/100 kcal/day) | 4.66 | 0-34.10 | 1.81-8.15 | 9.18 |
| Fungi and algae (gram/100 kcal/day) | 0 | 0-27.10 | 0-0.38 | 0.73 |
| Roots and tubers (gram/100 kcal/day) | 2.29 | 0-32.71 | 0-2.29 | 2.82 |
| Red meat other than pork (gram/100 kcal/day) | 0.01 | 0-29.08 | 0-0.76 | 1.25 |

**Table S7. Changes in cardiometabolic risk factors during follow-up associated with weighted Healthy Diet Score at baseline**

|  | **Healthy Diet Score** | | | | | P-trend* |
| --- | --- | --- | --- | --- | --- | --- |
|  | Quintile 1 | Quintile 2 | Quintile 3 | Quintile 4 | Quintile 5 |  |
| Change in BMI^†^ |  |  |  |  |  |  |
| Participants | 1082 | 1094 | 1096 | 1097 | 1100 |  |
| β (95% CI), Model 1^‡^ | 0 | -0.09 (-0.14, -0.04)^§, a^ | -0.10 (-0.15, -0.05)^a^ | -0.07 (-0.12, -0.02) | -0.09 (-0.14, -0.04) | 0.0045 |
| β (95% CI), Model 2 | 0 | -0.09 (-0.14, -0.04) | -0.10 (-0.15, -0.05)^a^ | -0.07 (-0.12, -0.02) | -0.09 (-0.14, -0.04)^a^ | 0.0034 |
| β (95% CI), Model 3 | 0 | -0.08 (-0.13, -0.03) | -0.10 (-0.15, -0.05)^a^ | -0.06 (-0.11, -0.01) | -0.07 (-0.12, -0.02) | 0.0257 |
| Change in WC |  |  |  |  |  |  |
| Participants | 1082 | 1090 | 1092 | 1091 | 1099 |  |
| β (95% CI), Model 1 | 0 | -0.01 (-0.05, 0.03) | -0.01 (-0.05, 0.02) | -0.01 (-0.05, 0.03) | -0.00 (-0.04, 0.04) | 0.99 |
| β (95% CI), Model 2 | 0 | -0.01 (-0.05, 0.02) | -0.02 (-0.06, 0.02) | -0.02 (-0.05, 0.02) | -0.00 (-0.04, 0.04) | 0.88 |
| β (95% CI), Model 3 | 0 | -0.01 (-0.05, 0.03) | -0.02 (-0.06, 0.02) | -0.02 (-0.05, 0.02) | -0.00 (-0.04, 0.04) | 0.76 |
| Change in PBF |  |  |  |  |  |  |
| Participants | 1040 | 1064 | 1066 | 1075 | 1072 |  |
| β (95% CI), Model 1 | 0 | -0.04 (-0.10, 0.02) | -0.08 (-0.14, -0.01) | -0.11 (-0.17, -0.05) | -0.15 (-0.21, -0.08)^a^ | <0.001 |
| β (95% CI), Model 2 | 0 | -0.05 (-0.11, 0.01) | -0.10 (-0.16, -0.03) | -0.13 (-0.19, -0.07)^a^ | -0.17 (-0.23, -0.11)^ab^ | <0.001 |
| β (95% CI), Model 3 | 0 | -0.05 (-0.11, 0.02) | -0.09 (-0.15, -0.03) | -0.13 (-0.19, -0.06)^a^ | -0.16 (-0.23, -0.10)^ab^ | <0.001 |
| Change in SBP |  |  |  |  |  |  |
| Participants | 1085 | 1092 | 1093 | 1090 | 1094 |  |
| β (95% CI), Model 1 | 0 | -0.06 (-0.14, 0.01) | -0.19 (-0.27, -0.11)^a^ | -0.31 (-0.39, -0.23)^ab^ | -0.47 (-0.55, -0.39)^abcd^ | <0.001 |
| β (95% CI), Model 2 | 0 | -0.07 (-0.14, 0.01) | -0.19 (-0.26, -0.11)^a^ | -0.30 (-0.38, -0.22)^ab^ | -0.43 (-0.51, -0.35)^abc^ | <0.001 |
| β (95% CI), Model 3 | 0 | -0.06 (-0.14, 0.02) | -0.18 (-0.26, -0.10)^a^ | -0.29 (-0.37, -0.21)^ab^ | -0.41 (-0.49, -0.33)^abc^ | <0.001 |
| Change in DBP |  |  |  |  |  |  |
| Participants | 1087 | 1094 | 1095 | 1091 | 1094 |  |
| β (95% CI), Model 1 | 0 | -0.10 (-0.18, -0.02) | -0.26 (-0.34, -0.18)^ab^ | -0.36 (-0.44, -0.28)^ab^ | -0.48 (-0.56, -0.40)^abc^ | <0.001 |
| β (95% CI), Model 2 | 0 | -0.11 (-0.19, -0.03) | -0.25 (-0.33, -0.17)^a^ | -0.36 (-0.44, -0.28)^ab^ | -0.46 (-0.55, -0.38)^abc^ | <0.001 |
| β (95% CI), Model 3 | 0 | -0.10 (-0.18, -0.03) | -0.25 (-0.33, -0.17)^a^ | -0.35 (-0.44, -0.27)^ab^ | -0.46 (-0.54, -0.37)^abc^ | <0.001 |
| Change in MAP |  |  |  |  |  |  |
| Participants | 1086 | 1092 | 1094 | 1089 | 1094 |  |
| β (95% CI), Model 1 | 0 | -0.09 (-0.17, -0.01) | -0.25 (-0.33, -0.17)^ab^ | -0.37 (-0.45, -0.28)^ab^ | -0.51 (-0.60, -0.43)^abcd^ | <0.001 |
| β (95% CI), Model 2 | 0 | -0.10 (-0.17, -0.02) | -0.24 (-0.32, -0.17)^ab^ | -0.36 (-0.44, -0.28)^ab^ | -0.49 (-0.57, -0.40)^abc^ | <0.001 |
| β (95% CI), Model 3 | 0 | -0.09 (-0.17, -0.01) | -0.24 (-0.32, -0.16)^ab^ | -0.35 (-0.43, -0.27)^ab^ | -0.47 (-0.55, -0.39)^abc^ | <0.001 |
| Change in TC |  |  |  |  |  |  |
| Participants | 1030 | 1016 | 1035 | 1029 | 1034 |  |
| β (95% CI), Model 1 | 0 | 0.02 (-0.04, 0.08) | 0.11 (0.05, 0.17)^a^ | 0.19 (0.13, 0.25)^ab^ | 0.29 (0.23, 0.35)^abc^ | <0.001 |
| β (95% CI), Model 2 | 0 | 0.00 (-0.06, 0.06) | 0.09 (0.03, 0.16) | 0.16 (0.10, 0.23)^ab^ | 0.27 (0.21, 0.33)^abc^ | <0.001 |
| β (95% CI), Model 3 | 0 | 0.00 (-0.06, 0.07) | 0.10 (0.04, 0.16) | 0.16 (0.09, 0.22)^ab^ | 0.25 (0.19, 0.32)^abc^ | <0.001 |
| Change in HDL-C |  |  |  |  |  |  |
| Participants | 1030 | 1014 | 1032 | 1032 | 1033 |  |
| β (95% CI), Model 1 | 0 | 0.02 (-0.06, 0.11) | 0.14 (0.06, 0.23) | 0.28 (0.20, 0.37)^ab^ | 0.49 (0.40, 0.58)^abcd^ | <0.001 |
| β (95% CI), Model 2 | 0 | 0.01 (-0.07, 0.10) | 0.12 (0.03, 0.20) | 0.25 (0.17, 0.34)^ab^ | 0.44 (0.35, 0.53)^abcd^ | <0.001 |
| β (95% CI), Model 3 | 0 | 0.01 (-0.07, 0.10) | 0.12 (0.04, 0.21) | 0.25 (0.16, 0.34)^ab^ | 0.43 (0.34, 0.52)^abcd^ | <0.001 |
| Change in LDL-C |  |  |  |  |  |  |
| Participants | 1029 | 1017 | 1036 | 1030 | 1034 |  |
| β (95% CI), Model 1 | 0 | -0.01 (-0.08, 0.05) | 0.07 (0.01, 0.14) | 0.30 (0.23, 0.36)^abc^ | 0.53 (0.46, 0.60)^abcd^ | <0.001 |
| β (95% CI), Model 2 | 0 | -0.02 (-0.08, 0.05) | 0.08 (0.01, 0.15) | 0.29 (0.23, 0.36)^abc^ | 0.53 (0.47, 0.60)^abcd^ | <0.001 |
| β (95% CI), Model 3 | 0 | -0.01 (-0.08, 0.05) | 0.08 (0.02, 0.15) | 0.28 (0.21, 0.35)^abc^ | 0.50 (0.43, 0.57)^abcd^ | <0.001 |
| Change in TG |  |  |  |  |  |  |
| Participants | 1030 | 1016 | 1036 | 1031 | 1035 |  |
| β (95% CI), Model 1 | 0 | 0.04 (-0.04, 0.12) | -0.02 (-0.10, 0.06) | -0.03 (-0.11, 0.05) | -0.08 (-0.16, -0.00) | 0.93 |
| β (95% CI), Model 2 | 0 | 0.04 (-0.04, 0.11) | -0.01 (-0.09, 0.07) | -0.03 (-0.11, 0.05) | -0.05 (-0.13, 0.03) | 0.85 |
| β (95% CI), Model 3 | 0 | 0.04 (-0.04, 0.11) | -0.01 (-0.09, 0.07) | -0.03 (-0.11, 0.05) | -0.07 (-0.15, 0.01) | 0.57 |
| Change in fasting glucose |  |  |  |  |  |  |
| Participants | 1028 | 1015 | 1037 | 1034 | 1033 |  |
| β (95% CI), Model 1 | 0 | -0.13 (-0.20, -0.07)^a^ | -0.25 (-0.32, -0.18)^ab^ | -0.23 (-0.29, -0.16)^a^ | -0.22 (-0.28, -0.15)^a^ | <0.001 |
| β (95% CI), Model 2 | 0 | -0.12 (-0.18, -0.05)^a^ | -0.23 (-0.29, -0.16)^ab^ | -0.20 (-0.27, -0.13)^a^ | -0.20 (-0.27, -0.14)^a^ | <0.001 |
| β (95% CI), Model 3 | 0 | -0.11 (-0.18, -0.05)^a^ | -0.23 (-0.29, -0.16)^ab^ | -0.20 (-0.27, -0.13)^a^ | -0.20 (-0.27, -0.14)^a^ | <0.001 |
| Change in insulin |  |  |  |  |  |  |
| Participants | 892 | 872 | 897 | 933 | 934 |  |
| β (95% CI), Model 1 | 0 | -0.01 (-0.14, 0.12) | -0.01 (-0.13, 0.12) | -0.32 (-0.45, -0.19)^abc^ | -0.63 (-0.76, -0.50)^abcd^ | <0.001 |
| β (95% CI), Model 2 | 0 | -0.00 (-0.13, 0.12) | 0.01 (-0.11, 0.14) | -0.28 (-0.40, -0.15)^abc^ | -0.53 (-0.66, -0.41)^abcd^ | <0.001 |
| β (95% CI), Model 3 | 0 | 0.00 (-0.12, 0.13) | 0.02 (-0.10, 0.15) | -0.26 (-0.39, -0.14)^bc^ | -0.51 (-0.63, -0.38)^abcd^ | <0.001 |
| Change in HOMA-IR |  |  |  |  |  |  |
| Participants | 892 | 871 | 897 | 933 | 933 |  |
| β (95% CI), Model 1 | 0 | -0.04 (-0.17, 0.08) | -0.06 (-0.19, 0.06) | -0.36 (-0.49, -0.24)^abc^ | -0.66 (-0.78, -0.53)^abcd^ | <0.001 |
| β (95% CI), Model 2 | 0 | -0.03 (-0.15, 0.09) | -0.04 (-0.17, 0.08) | -0.32 (-0.44, -0.20)^abc^ | -0.56 (-0.69, -0.44)^abcd^ | <0.001 |
| β (95% CI), Model 3 | 0 | -0.02 (-0.14, 0.10) | -0.03 (-0.15, 0.09) | -0.31 (-0.43, -0.18)^abc^ | -0.54 (-0.66, -0.41)^abcd^ | <0.001 |
| Change in CMRS |  |  |  |  |  |  |
| Participants | 921 | 922 | 952 | 938 | 958 |  |
| β (95% CI), Model 1 | 0 | -0.16 (-0.36, 0.04) | -0.60 (-0.80, -0.40)^ab^ | -0.93 (-1.13, -0.73)^ab^ | -1.28 (-1.48, -1.08)^abcd^ | <0.001 |
| β (95% CI), Model 2 | 0 | -0.14 (-0.33, 0.04) | -0.56 (-0.75, -0.37)^ab^ | -0.85 (-1.05, -0.66)^ab^ | -1.16 (-1.35, -0.97)^abc^ | <0.001 |
| β (95% CI), Model 3 | 0 | -0.13 (-0.32, 0.05) | -0.54 (-0.73, -0.36)^ab^ | -0.83 (-1.03, -0.64)^ab^ | -1.13 (-1.32, -0.94)^abc^ | <0.0001 |

BMI, body mass index; CMRS, cardiometabolic risk score; DBP, diastolic blood pressure; HOMA-IR, homeostatic model assessment of insulin resistance; HDL-C, high-density lipoprotein cholesterol; LDL-C, low-density lipoprotein cholesterol; MAP, mean arterial pressure; SBP, systolic blood pressure; SE, standard error; TC, total cholesterol; TG, triglyceride.

*GLM was used to estimate beta coefficients (β) and 95% CIs of cardiometabolic risk factors between quintiles. Benjamin-Hochberg's procedure was used to control the false discovery rate at level 5% for multiple comparisons with the P-value cut-off point of significance was 0.0433 for HDS and changes in CMR factors (Model 3).

^†^Changes in CMR factors were calculated by subtracting the results at baseline from those at follow-up.

^‡^Model 1 was adjusted for classes in school as clustering effects and characteristics of individuals including age, sex, and corresponding CMR factor at baseline as fixed effects; Model 2 was adjusted for Model 1 plus puberty, grade, intervention, BMI, physical activity, and intake of energy, fiber, vegetable, fruit, pork, legumes, and nuts at baseline as fixed effects; Model 3 was adjusted for Model 2 plus birthweight, household income, mother’s education, father’s education, mother’s BMI, and father’s BMI as fixed effects.

^§^All these data are β (95% CI) of changes in CMR factors.

^abcd^Bonferroni Post-hoc test was used to examine the difference between every two quintiles of the healthy diet score with ^a^ indicating significance compared with quintile 1, ^b^ indicating significance compared with quintile 2, ^c^ indicating significance compared with quintile 3, and ^d^ indicating significance compared with quintile 4.

**Table S8. Changes in cardiometabolic risk factors during follow-up associated with change in healthy dietary score***

|  | Coefficient | P-value |
| --- | --- | --- |
| Change in BMI |  |  |
| Model 1^†^ | -0.0130 | 0.0517 |
| Model 2^‡^ | -0.0158 | 0.0190 |
| Model 3^§^ | -0.0146 | 0.0304 |
| Change in WC |  |  |
| Model 1 | -0.0032 | 0.56 |
| Model 2 | -0.0040 | 0.46 |
| Model 3 | -0.0033 | 0.55 |
| Change in PBF |  |  |
| Model 1 | -0.0007 | 0.94 |
| Model 2 | -0.0046 | 0.61 |
| Model 3 | -0.0039 | 0.66 |
| Change in SBP |  |  |
| Model 1 | -0.0700 | <0.0001 |
| Model 2 | -0.0633 | <0.0001 |
| Model 3 | -0.0623 | <0.0001 |
| Change in DBP |  |  |
| Model 1 | -0.0537 | <0.0001 |
| Model 2 | -0.0492 | <0.0001 |
| Model 3 | -0.0492 | <0.0001 |
| Change in MAP |  |  |
| Model 1 | -0.0644 | <0.0001 |
| Model 2 | -0.0586 | <0.0001 |
| Model 3 | -0.0582 | <0.0001 |
| Change in TC |  |  |
| Model 1 | 0.0216 | 0.0143 |
| Model 2 | 0.0118 | 0.18 |
| Model 3 | 0.0128 | 0.15 |
| Change in HDL-C |  |  |
| Model 1 | 0.0368 | 0.0041 |
| Model 2 | 0.0241 | 0.0587 |
| Model 3 | 0.0235 | 0.0649 |
| Change in LDL-C |  |  |
| Model 1 | 0.0559 | <0.0001 |
| Model 2 | 0.04863 | <0.0001 |
| Model 3 | 0.04774 | <0.0001 |
| Change in TG |  |  |
| Model 1 | -0.00386 | 0.73 |
| Model 2 | -0.00286 | 0.80 |
| Model 3 | -0.00307 | 0.79 |
| Change in fasting glucose |  |  |
| Model 1 | -0.02296 | 0.0149 |
| Model 2 | -0.02383 | 0.0131 |
| Model 3 | -0.02448 | 0.0110 |
| Change in insulin |  |  |
| Model 1 | -0.08275 | <0.0001 |
| Model 2 | -0.06839 | 0.0002 |
| Model 3 | -0.06701 | 0.0002 |
| Change in HOMA-IR |  |  |
| Model 1 | -0.08583 | <0.0001 |
| Model 2 | -0.07197 | <0.0001 |
| Model 3 | -0.07054 | <0.0001 |
| Change in CMRS |  |  |
| Model 1 | -0.12922 | <0.0001 |
| Model 2 | -0.11863 | <0.0001 |
| Model 3 | -0.11763 | <0.0001 |

BMI, body mass index; CMRS, cardiometabolic risk score; DBP, diastolic blood pressure; HOMA-IR, homeostatic model assessment of insulin resistance; HDL-C, high-density lipoprotein cholesterol; LDL-C, low-density lipoprotein cholesterol; MAP, mean arterial pressure; SBP, systolic blood pressure; SE, standard error; TC, total cholesterol; TG, triglyceride.

*Change in healthy dietary score refers to a change between baseline and follow-up (subtracting HDS at baseline from that at follow-up) and improved HDS indicates an increase in HDS.

^†^Model 1 was adjusted for classes in school as clustering effects and characteristics of individuals including age, sex, and corresponding CMR factor at baseline as fixed effects.

^‡^Model 2 was adjusted for Model 1 plus puberty, grade, intervention, BMI, physical activity, and intake of energy, fiber, vegetable, fruit, pork, legumes, and nuts at baseline as fixed effects.

^§^Model 3 was adjusted for Model 2 plus birthweight, household income, mother’s education, father’s education, mother’s BMI, and father’s BMI as fixed effects.

**Table S9. Changes in cardiometabolic risk factors during follow-up associated with healthy dietary score at baseline in the control group**

|  | **Healthy Diet Score** | | | | | | P-trend |
| --- | --- | --- | --- | --- | --- | --- | --- |
|  | ≤3 | 4 | 5 | 6 | 7 | ≥8 |  |
| Change in BMI |  |  |  |  |  |  |  |
| Participants | 282 | 435 | 683 | 713 | 359 | 115 |  |
| Mean ± SE, Model 1^†^ | 0.21 ± 0.04 | 0.07 ± 0.03 | 0.08 ± 0.03 | 0.07 ± 0.03 | 0.13 ± 0.03 | 0.06 ± 0.06 | 0.14 |
| Mean ± SE, Model 2^‡^ | 0.20 ± 0.04 | 0.09 ± 0.03 | 0.11 ± 0.02 | 0.09 ± 0.03 | 0.16 ± 0.03 | 0.10 ± 0.06 | 0.46 |
| Mean ± SE, Model 3^§^ | 0.26 ± 0.05 | 0.15 ± 0.04 | 0.16 ± 0.04 | 0.15 ± 0.04 | 0.22 ± 0.05 | 0.16 ± 0.06 | 0.55 |
| Change in WC |  |  |  |  |  |  |  |
| Participants | 285 | 433 | 683 | 711 | 359 | 116 |  |
| Mean ± SE, Model 1 | 0.28 ± 0.03 | 0.19 ± 0.03 | 0.20 ± 0.02 | 0.20 ± 0.02 | 0.21 ± 0.03 | 0.14 ± 0.05 | 0.08 |
| Mean ± SE, Model 2 | 0.29 ± 0.03 | 0.22 ± 0.02 | 0.23 ± 0.02 | 0.23 ± 0.02 | 0.25 ± 0.03 | 0.18 ± 0.04 | 0.27 |
| Mean ± SE, Model 3 | 0.31 ± 0.04 | 0.24 ± 0.03 | 0.25 ± 0.03 | 0.25 ± 0.03 | 0.27 ± 0.03 | 0.20 ± 0.05 | 0.22 |
| Change in PBF |  |  |  |  |  |  |  |
| Participants | 270 | 421 | 670 | 694 | 350 | 113 |  |
| Mean ± SE, Model 1 | 0.39 ± 0.05 | 0.31 ± 0.04 | 0.24 ± 0.03 | 0.21 ± 0.03^a^ | 0.15 ± 0.04^a^ | 0.14 ± 0.07 | <0.0001 |
| Mean ± SE, Model 2 | 0.37 ± 0.05 | 0.29 ± 0.04 | 0.22 ± 0.03 | 0.21 ± 0.03 | 0.15 ± 0.04^a^ | 0.15 ± 0.07 | <0.0001 |
| Mean ± SE, Model 3 | 0.35 ± 0.06 | 0.28 ± 0.05 | 0.21 ± 0.05 | 0.20 ± 0.05 | 0.14 ± 0.06^a^ | 0.13 ± 0.07 | <0.0001 |
| Change in SBP |  |  |  |  |  |  |  |
| Participants | 285 | 434 | 688 | 705 | 360 | 116 |  |
| Mean ± SE, Model 1 | 0.26 ± 0.07 | 0.14 ± 0.06 | 0.12 ± 0.05 | -0.04 ± 0.05^a^ | -0.09 ± 0.06^a^ | -0.31 ± 0.10^abc^ | <0.0001 |
| Mean ± SE, Model 2 | 0.21 ± 0.07 | 0.13 ± 0.06 | 0.13 ± 0.05 | -0.01 ± 0.05 | -0.07 ± 0.06^a^ | -0.26 ± 0.10^abc^ | <0.0001 |
| Mean ± SE, Model 3 | 0.07 ± 0.08 | -0.01 ± 0.08 | -0.02 ± 0.07 | -0.14 ± 0.07 | -0.21 ± 0.08^a^ | -0.42 ± 0.11^abc^ | <0.0001 |
| Change in DBP |  |  |  |  |  |  |  |
| Participants | 285 | 434 | 688 | 707 | 359 | 116 |  |
| Mean ± SE, Model 1 | 0.17 ± 0.07 | 0.13 ± 0.06 | 0.03 ± 0.05 | -0.14 ± 0.05^ab^ | -0.14 ± 0.06^ab^ | -0.36 ± 0.10^abc^ | <0.0001 |
| Mean ± SE, Model 2 | 0.13 ± 0.07 | 0.12 ± 0.06 | 0.04 ± 0.05 | -0.12 ± 0.05^ab^ | -0.13 ± 0.06^b^ | -0.33 ± 0.10^abc^ | <0.0001 |
| Mean ± SE, Model 3 | 0.02 ± 0.08 | 0.01 ± 0.07 | -0.07 ± 0.07 | -0.23 ± 0.07^b^ | -0.24 ± 0.08^b^ | -0.46 ± 0.11^abc^ | <0.0001 |
| Change in MAP |  |  |  |  |  |  |  |
| Participants | 285 | 434 | 688 | 705 | 359 | 116 |  |
| Mean ± SE, Model 1 | 0.22 ± 0.07 | 0.15 ± 0.06 | 0.07 ± 0.05 | -0.11 ± 0.05^ab^ | -0.13 ± 0.06^ab^ | -0.39 ± 0.10^abc^ | <0.0001 |
| Mean ± SE, Model 2 | 0.17 ± 0.07 | 0.14 ± 0.06 | 0.08 ± 0.05 | -0.09 ± 0.05^ab^ | -0.12 ± 0.06^ab^ | -0.34 ± 0.10^abc^ | <0.0001 |
| Mean ± SE, Model 3 | 0.04 ± 0.09 | -0.00 ± 0.08 | -0.05 ± 0.07 | -0.22 ± 0.07^ab^ | -0.25 ± 0.08^ab^ | -0.49 ± 0.11^abc^ | <0.0001 |
| Change in TC |  |  |  |  |  |  |  |
| Participants | 266 | 402 | 642 | 676 | 333 | 107 |  |
| Mean ± SE, Model 1 | 0.01 ± 0.06 | 0.05 ± 0.06 | 0.05 ± 0.06 | 0.13 ± 0.06 | 0.12 ± 0.06 | 0.12 ± 0.08 | 0.0084 |
| Mean ± SE, Model 2 | 0.02 ± 0.06 | 0.06 ± 0.06 | 0.06 ± 0.06 | 0.14 ± 0.06 | 0.13 ± 0.06 | 0.14 ± 0.08 | 0.0089 |
| Mean ± SE, Model 3 | -0.04 ± 0.07 | 0.00 ± 0.07 | 0.01 ± 0.06 | 0.08 ± 0.07 | 0.07 ± 0.07 | 0.09 ± 0.09 | 0.0095 |
| Change in HDL-C |  |  |  |  |  |  |  |
| Participants | 266 | 402 | 640 | 680 | 334 | 107 |  |
| Mean ± SE, Model 1 | 0.36 ± 0.09 | 0.48 ± 0.08 | 0.53 ± 0.08 | 0.62 ± 0.08^a^ | 0.72 ± 0.08^a^ | 0.59 ± 0.11 | <0.0001 |
| Mean ± SE, Model 2 | 0.38 ± 0.09 | 0.47 ± 0.08 | 0.50 ± 0.08 | 0.56 ± 0.08 | 0.68 ± 0.09^a^ | 0.57 ± 0.11 | 0.0002 |
| Mean ± SE, Model 3 | 0.33 ± 0.10 | 0.43 ± 0.09 | 0.46 ± 0.09 | 0.51 ± 0.09 | 0.64 ± 0.10^a^ | 0.53 ± 0.12 | 0.0003 |
| Change in LDL-C |  |  |  |  |  |  |  |
| Participants | 266 | 402 | 643 | 677 | 333 | 107 |  |
| Mean ± SE, Model 1 | 0.10 ± 0.06 | 0.11 ± 0.06 | 0.09 ± 0.05 | 0.24 ± 0.05^c^ | 0.30 ± 0.06^c^ | 0.38 ± 0.08^c^ | <0.0001 |
| Mean ± SE, Model 2 | 0.14 ± 0.06 | 0.16 ± 0.05 | 0.15 ± 0.05 | 0.30 ± 0.05^c^ | 0.34 ± 0.05^c^ | 0.44 ± 0.08^abc^ | <0.0001 |
| Mean ± SE, Model 3 | 0.08 ± 0.07 | 0.10 ± 0.06 | 0.09 ± 0.06 | 0.22 ± 0.06 | 0.26 ± 0.07 | 0.36 ± 0.09^c^ | <0.0001 |
| Change in TG |  |  |  |  |  |  |  |
| Participants | 266 | 402 | 641 | 680 | 334 | 108 |  |
| Mean ± SE, Model 1 | 0.16 ± 0.07 | 0.16 ± 0.07 | 0.12 ± 0.06 | 0.04 ± 0.06 | -0.09 ± 0.07^b^ | -0.08 ± 0.10 | <0.0001 |
| Mean ± SE, Model 2 | 0.10 ± 0.07 | 0.16 ± 0.07 | 0.14 ± 0.06 | 0.07 ± 0.06 | -0.04 ± 0.07 | -0.04 ± 0.10 | 0.0039 |
| Mean ± SE, Model 3 | 0.06 ± 0.09 | 0.10 ± 0.08 | 0.10 ± 0.07 | 0.03 ± 0.08 | -0.09 ± 0.08 | -0.10 ± 0.11 | 0.0034 |
| Change in fasting glucose |  |  |  |  |  |  |  |
| Participants | 266 | 401 | 643 | 680 | 334 | 106 |  |
| Mean ± SE, Model 1 | 0.43 ± 0.09 | 0.44 ± 0.08 | 0.43 ± 0.08 | 0.45 ± 0.08 | 0.44 ± 0.08 | 0.57 ± 0.10 | 0.29 |
| Mean ± SE, Model 2 | 0.46 ± 0.09 | 0.45 ± 0.08 | 0.43 ± 0.08 | 0.46 ± 0.08 | 0.44 ± 0.08 | 0.58 ± 0.10 | 0.49 |
| Mean ± SE, Model 3 | 0.47 ± 0.10 | 0.45 ± 0.09 | 0.44 ± 0.09 | 0.46 ± 0.09 | 0.44 ± 0.09 | 0.56 ± 0.11 | 0.67 |
| Change in insulin |  |  |  |  |  |  |  |
| Participants | 246 | 360 | 565 | 599 | 306 | 98 |  |
| Mean ± SE, Model 1 | 0.05 ± 0.11 | -0.17 ± 0.10 | -0.18 ± 0.09 | -0.41 ± 0.08^a^ | -0.64 ± 0.10^abc^ | -0.63 ± 0.15^a^ | <0.0001 |
| Mean ± SE, Model 2 | -0.03 ± 0.10 | -0.21 ± 0.09 | -0.22 ± 0.08 | -0.41 ± 0.08 | -0.62 ± 0.10^abc^ | -0.58 ± 0.15 | <0.0001 |
| Mean ± SE, Model 3 | -0.01 ± 0.13 | -0.22 ± 0.12 | -0.21 ± 0.11 | -0.38 ± 0.11 | -0.59 ± 0.12^ac^ | -0.56 ± 0.17 | <0.0001 |
| Change in HOMA-IR |  |  |  |  |  |  |  |
| Participants | 246 | 360 | 565 | 599 | 306 | 97 |  |
| Mean ± SE, Model 1 | 0.16 ± 0.11 | -0.05 ± 0.09 | -0.07 ± 0.08 | -0.29 ± 0.08^a^ | -0.51 ± 0.10^abc^ | -0.48 ± 0.15^a^ | <0.0001 |
| Mean ± SE, Model 2 | 0.09 ± 0.10 | -0.09 ± 0.09 | -0.11 ± 0.08 | -0.28 ± 0.08 | -0.49 ± 0.09^abc^ | -0.43 ± 0.14 | <0.0001 |
| Mean ± SE, Model 3 | 0.11 ± 0.13 | -0.09 ± 0.11 | -0.09 ± 0.10 | -0.25 ± 0.11 | -0.45 ± 0.12^ac^ | -0.41 ± 0.16 | <0.0001 |
| Change in CMRS |  |  |  |  |  |  |  |
| Participants | 232 | 354 | 591 | 625 | 312 | 102 |  |
| Mean ± SE, Model 1 | 0.65 ± 0.22 | 0.23 ± 0.20 | 0.25 ± 0.19 | -0.05 ± 0.19^a^ | -0.36 ± 0.21^ac^ | -0.32 ± 0.27^a^ | <0.0001 |
| Mean ± SE, Model 2 | 0.58 ± 0.20 | 0.26 ± 0.19 | 0.33 ± 0.17 | 0.09 ± 0.18 | -0.22 ± 0.19^ac^ | -0.13 ± 0.26 | <0.0001 |
| Mean ± SE, Model 3 | 0.59 ± 0.24 | 0.24 ± 0.22 | 0.34 ± 0.21 | 0.10 ± 0.21 | -0.22 ± 0.23^ac^ | -0.15 ± 0.28 | <0.0001 |

BMI, body mass index; CMRS, cardiometabolic risk score; DBP, diastolic blood pressure; HOMA-IR, homeostatic model assessment of insulin resistance; HDL-C, high-density lipoprotein cholesterol; LDL-C, low-density lipoprotein cholesterol; MAP, mean arterial pressure; SBP, systolic blood pressure; SE, standard error; TC, total cholesterol; TG, triglyceride.

^†^Model 1 was adjusted for classes in school as clustering effects and characteristics of individuals including age, sex, and corresponding CMR factor at baseline as fixed effects.

^‡^Model 2 was adjusted for Model 1 plus puberty, grade, intervention, BMI, physical activity, and intake of energy, fiber, vegetable, fruit, pork, legumes, and nuts at baseline as fixed effects.

^§^Model 3 was adjusted for Model 2 plus birthweight, household income, mother’s education, father’s education, mother’s BMI, and father’s BMI as fixed effects.

^abcd^Bonferroni Post-hoc test was used to examine the difference between each two groups of healthy dietary score with ^a^ indicating significance compared with HDS≤3, ^b^ indicating significance compared with HDS=4, and ^c^ indicating significance compared with HDS=5. The comparisons with HDS=6 and HDS=7 were also conducted, but no significant associations were found.

**Table S10. Validation of healthy dietary score in children from China Nutrition and Health Survey**

|  | **Healthy dietary score** | | | | | P-trend |
| --- | --- | --- | --- | --- | --- | --- |
|  | ≤4 | 5 | 6 | 7 | ≥8 |  |
| BMI at baseline |  |  |  |  |  |  |
| Participants | 232 | 357 | 2602 | 1244 | 95 |  |
| Mean ± SE, Model 1^†^ | 0.14 ± 0.07 | 0.04 ± 0.06 | -0.26 ± 0.04 | -0.26 ± 0.04 | -0.26 ± 0.10 | <0.0001 |
| Mean ± SE, Model 2^‡^ | 0.25 ± 0.14 | 0.16 ± 0.14 | -0.12 ± 0.13 | -0.13 ± 0.13 | -0.16 ± 0.16 | <0.0001 |
| WC at baseline |  |  |  |  |  |  |
| Participants | 213 | 326 | 1636 | 843 | 92 |  |
| Mean ± SE, Model 1 | 0.33 ± 0.06 | 0.17 ± 0.05 | -0.04 ± 0.03 | -0.06 ± 0.04 | -0.06 ± 0.10 | <0.0001 |
| Mean ± SE, Model 2 | 0.60 ± 0.14 | 0.45 ± 0.14 | 0.26 ± 0.13 | 0.22 ± 0.13 | 0.19 ± 0.15 | <0.0001 |
| SBP at baseline |  |  |  |  |  |  |
| Participants | 189 | 306 | 2461 | 1170 | 83 |  |
| Mean ± SE, Model 1 | -0.35 ± 0.08 | -0.54 ± 0.07 | -0.74 ± 0.03 | -0.68 ± 0.04 | -0.41 ± 0.12 | 0.0265 |
| Mean ± SE, Model 2 | 0.26 ± 0.17 | 0.08 ± 0.17 | -0.09 ± 0.16 | -0.04 ± 0.16 | 0.18 ± 0.19 | 0.0415 |
| DBP at baseline |  |  |  |  |  |  |
| Participants | 189 | 306 | 2457 | 1172 | 83 |  |
| Mean ± SE, Model 1 | 0.27 ± 0.07 | 0.06 ± 0.06 | -0.29 ± 0.02 | -0.25 ± 0.03 | -0.07 ± 0.11 | <0.0001 |
| Mean ± SE, Model 2 | 0.64 ± 0.16 | 0.44 ± 0.15 | 0.14 ± 0.14 | 0.17 ± 0.14 | 0.29 ± 0.18 | <0.0001 |
| MAP at baseline |  |  |  |  |  |  |
| Participants | 189 | 306 | 2457 | 1170 | 83 |  |
| Mean ± SE, Model 1 | 0.04 ± 0.08 | -0.18 ± 0.06 | -0.51 ± 0.02 | -0.45 ± 0.03 | -0.22 ± 0.12 | <0.0001 |
| Mean ± SE, Model 2 | 0.55 ± 0.17 | 0.34 ± 0.16 | 0.06 ± 0.15 | 0.10 ± 0.15 | 0.27 ± 0.19 | <0.0001 |
| Change in BMI |  |  |  |  |  |  |
| Participants | 215 | 352 | 2491 | 1185 | 87 |  |
| Mean ± SE, Model 1 | 0.49 ± 0.06 | 0.40 ± 0.05 | 0.49 ± 0.02 | 0.49 ± 0.03 | 0.38 ± 0.09 | 0.85 |
| Mean ± SE, Model 2 | 0.51 ± 0.13 | 0.41 ± 0.13 | 0.49 ± 0.12 | 0.47 ± 0.12 | 0.39 ± 0.15 | 0.82 |
| Change in WC |  |  |  |  |  |  |
| Participants | 197 | 317 | 1513 | 782 | 87 |  |
| Mean ± SE, Model 1 | 0.63 ± 0.07 | 0.53 ± 0.06 | 0.57 ± 0.04 | 0.60 ± 0.04 | 0.44 ± 0.10 | 0.74 |
| Mean ± SE, Model 2 | 0.57 ± 0.15 | 0.47 ± 0.15 | 0.51 ± 0.14 | 0.53 ± 0.14 | 0.40 ± 0.17 | 0.66 |
| Change SBP |  |  |  |  |  |  |
| Participants | 160 | 278 | 2327 | 1085 | 78 |  |
| Mean ± SE, Model 1 | 0.48 ± 0.09 | 0.30 ± 0.07 | 0.48 ± 0.03 | 0.49 ± 0.04 | 0.31 ± 0.13 | 0.45 |
| Mean ± SE, Model 2 | 0.43 ± 0.19 | 0.23 ± 0.18 | 0.44 ± 0.16 | 0.44 ± 0.17 | 0.25 ± 0.21 | 0.45 |
| Change in DBP |  |  |  |  |  |  |
| Participants | 160 | 278 | 2324 | 1086 | 78 |  |
| Mean ± SE, Model 1 | 0.51 ± 0.08 | 0.40 ± 0.06 | 0.56 ± 0.02 | 0.61 ± 0.03 | 0.40 ± 0.11 | 0.05 |
| Mean ± SE, Model 2 | 0.48 ± 0.16 | 0.35 ± 0.15 | 0.55 ± 0.14 | 0.59 ± 0.14 | 0.35 ± 0.18 | 0.05 |
| Change in MAP |  |  |  |  |  |  |
| Participants | 160 | 278 | 2323 | 1084 | 78 |  |
| Mean ± SE, Model 1 | 0.55 ± 0.09 | 0.38 ± 0.07 | 0.58 ± 0.02 | 0.61 ± 0.03 | 0.40 ± 0.13 | 0.14 |
| Mean ± SE, Model 2 | 0.47 ± 0.18 | 0.29 ± 0.18 | 0.54 ± 0.16 | 0.55 ± 0.16 | 0.32 ± 0.20 | 0.13 |

BMI, body mass index; DBP, diastolic blood pressure; MAP, mean arterial pressure; SBP, systolic blood pressure; SE, standard error.

^†^Model 1 was adjusted age and sex.

^‡^Model 2 was adjusted for Model 1 plus education and BMI.

**Table S11. Red meat other than pork and other food items**

|  | Red meat other than pork  (gram/100 kcal/day) | | P-value* |
| --- | --- | --- | --- |
|  | >0.01 | ≦0.01 | |
| Rice (gram/100 kcal/day) | 13.41 ± 9.83 | 13.99 ± 10.68 | 0.08 |
| Wheat (gram/100 kcal/day) | 4.39 ± 4.85 | 4.67 ± 5.11 | 0.08 |
| Other cereals (gram/100 kcal/day) | 0.70 ± 1.85 | 0.77 ± 2.14 | 0.31 |
| Roots and tubers (gram/100 kcal/day) | 1.94 ± 3.16 | 1.82 ± 3.29 | 0.27 |
| Nuts and legumes (gram/100 kcal/day) | 2.15 ± 4.23 | 2.52 ± 5.11 | 0.0182 |
| Deep color vegetables (gram/100 kcal/day) | 4.52 ± 5.35 | 4.48 ± 5.60 | 0.80 |
| Light color vegetables (gram/100 kcal/day) | 7.45 ± 7.33 | 8.06 ± 8.23 | 0.0158 |
| Pickled vegetables (gram/100 kcal/day) | 0.12 ± 0.64 | 0.15 ± 0.96 | 0.22 |
| Fruits (gram/100 kcal/day) | 5.26 ± 9.25 | 5.52 ± 9.50 | 0.38 |
| **Pork (gram/100 kcal/day)** | **3.85 ± 3.46** | **4.42 ± 3.97** | **<0.0001** |
| Offal (gram/100 kcal/day) | 0.17 ± 0.86 | 0.14 ± 0.89 | 0.21 |
| Poultry (gram/100 kcal/day) | 2.54 ± 3.63 | 2.64 ± 4.37 | 0.49 |
| **Milk (gram/100 kcal/day)** | **8.20 ± 9.55** | **9.20 ± 10.96** | **0.0028** |
| Eggs (gram/100 kcal/day) | 2.81 ± 2.91 | 3.09 ± 3.44 | 0.0068 |
| Fish (gram/100 kcal/day) | 2.61 ± 4.19 | 2.57 ± 4.63 | 0.82 |
| Fast foods (gram/100 kcal/day) | 0.18 ± 1.02 | 0.15 ± 0.93 | 0.30 |
| Dairy product (gram/100 kcal/day) | 0.02 ± 0.26 | 0.03 ± 0.42 | 0.38 |
| Dessert (gram/100 kcal/day) | 0.75 ± 1.88 | 0.79 ± 1.87 | 0.44 |
| Candy and sugar (gram/100 kcal/day) | 0.79 ± 1.91 | 0.70 ± 1.74 | 0.10 |
| Fried foods (gram/100 kcal/day) | 0.71 ± 1.80 | 0.79 ± 2.03 | 0.21 |
| Catsup and other sources (gram/100 kcal/day) | 0.02 ± 0.41 | 0.04 ± 0.46 | 0.25 |
| Fungi and algae (gram/100 kcal/day) | 0.51 ± 1.15 | 0.50 ± 1.20 | 0.79 |
| Sugar-sweetened beverages (gram/100 kcal/day) | 2.05 ± 5.52 | 2.08 ± 5.28 | 0.85 |
| Refined grains (gram/100 kcal/day) | 5.25 ± 6.84 | 4.86 ± 6.10 | 0.0463 |
| Yogurt (gram/100 kcal/day) | 1.26 ± 4.12 | 1.25 ± 4.03 | 0.92 |

*T-test was used to examine the difference of the intake of other foods between children with high and low red meat other than pork intake. P-value<0.006 was considered statistically significant after controlling the false discovery rate at level of 5% using Benjamin-Hochberg's procedure.

**Table S12. Rice and other food items**

|  | Rice intake (gram/100 kcal/day) | |  |
| --- | --- | --- | --- |
|  | >5.99 | ≦5.99 | P-value* |
| Rice (gram/100 kcal/day) | 23.46 ± 9.72 | 7.30 ± 3.83 | <0.0001 |
| Wheat (gram/100 kcal/day) | 2.93 ± 4.11 | 5.75 ± 5.30 | <0.0001 |
| Other cereals (gram/100 kcal/day) | 0.53 ± 1.70 | 0.90 ± 2.28 | <0.0001 |
| Roots and tubers (gram/100 kcal/day) | 1.69 ± 3.55 | 1.96 ± 3.04 | 0.0025 |
| Nuts and legumes (gram/100 kcal/day) | 2.36 ± 5.46 | 2.48 ± 4.51 | 0.36 |
| Deep color vegetables (gram/100 kcal/day) | 5.48 ± 6.30 | 3.81 ± 4.83 | <0.0001 |
| Light color vegetables (gram/100 kcal/day) | 8.30 ± 9.05 | 7.66 ± 7.23 | 0.0033 |
| Pickled vegetables (gram/100 kcal/day) | 0.16 ± 1.05 | 0.13 ± 0.77 | 0.23 |
| Fruits (gram/100 kcal/day) | 4.76 ± 9.15 | 5.93 ± 9.61 | <0.0001 |
| Pork (gram/100 kcal/day) | 4.19 ± 3.91 | 4.35 ± 3.83 | 0.15 |
| Red meat other than pork (gram/100 kcal/day) | 0.92 ± 2.28 | 0.92 ± 2.24 | 0.97 |
| Offal (gram/100 kcal/day) | 0.14 ± 0.81 | 0.15 ± 0.93 | 0.50 |
| Poultry (gram/100 kcal/day) | 3.06 ± 4.64 | 2.31 ± 3.86 | <0.0001 |
| Milk (gram/100 kcal/day) | 8.96 ± 11.65 | 8.97 ± 9.91 | 0.99 |
| Eggs (gram/100 kcal/day) | 3.04 ± 3.60 | 3.01 ± 3.12 | 0.73 |
| Fish (gram/100 kcal/day) | 3.34 ± 4.95 | 2.06 ± 4.13 | <0.0001 |
| Fast foods (gram/100 kcal/day) | 0.10 ± 0.87 | 0.19 ± 1.00 | 0.0013 |
| Dairy product (gram/100 kcal/day) | 0.02 ± 0.18 | 0.04 ± 0.48 | 0.0454 |
| Dessert (gram/100 kcal/day) | 0.56 ± 1.70 | 0.93 ± 1.97 | <0.0001 |
| Candy and sugar (gram/100 kcal/day) | 0.81 ± 2.00 | 0.66 ± 1.61 | 0.0015 |
| Fried foods (gram/100 kcal/day) | 0.37 ± 1.43 | 1.04 ± 2.24 | <0.0001 |
| Catsup and other sources (gram/100 kcal/day) | 0.05 ± 0.58 | 0.02 ± 0.34 | 0.0302 |
| Fungi and algae (gram/100 kcal/day) | 0.35 ± 0.98 | 0.60 ± 1.29 | <0.0001 |
| Sugar-sweetened beverages (gram/100 kcal/day) | 1.47 ± 4.89 | 2.48 ± 5.58 | <0.0001 |
| Refined grains (gram/100 kcal/day) | 6.11 ± 7.56 | 4.16 ± 5.10 | <0.0001 |
| Yogurt (gram/100 kcal/day) | 0.96 ± 3.75 | 1.45 ± 4.23 | <0.0001 |

*T-test was used to examine the difference of the intake of other foods between children with high and low rice intake. P-value<0.0346 was considered statistically significant after controlling the false discovery rate at level of 5% using Benjamin-Hochberg's procedure.

**Table S13. Wheat and other food items**

|  | Wheat intake (gram/100 kcal/day) | |  |
| --- | --- | --- | --- |
|  | >4.66 | ≦4.66 | P-value* |
| Rice (gram/100 kcal/day) | 9.92 ± 7.45 | 16.33 ± 11.33 | <0.0001 |
| Wheat (gram/100 kcal/day) | 9.66 ± 4.56 | 1.43 ± 1.49 | <0.0001 |
| Other cereals (gram/100 kcal/day) | 0.93 ± 2.23 | 0.64 ± 1.96 | <0.0001 |
| Roots and tubers (gram/100 kcal/day) | 2.12 ± 3.37 | 1.68 ± 3.18 | <0.0001 |
| Nuts and legumes (gram/100 kcal/day) | 2.71 ± 5.53 | 2.26 ± 4.48 | 0.0009 |
| Deep color vegetables (gram/100 kcal/day) | 3.72 ± 4.75 | 4.97 ± 5.93 | <0.0001 |
| Light color vegetables (gram/100 kcal/day) | 8.05 ± 7.68 | 7.83 ± 8.24 | 0.32 |
| Pickled vegetables (gram/100 kcal/day) | 0.09 ± 0.53 | 0.18 ± 1.06 | 0.0001 |
| Fruits (gram/100 kcal/day) | 5.90 ± 9.56 | 5.18 ± 9.36 | 0.0055 |
| Pork (gram/100 kcal/day) | 4.05 ± 3.76 | 4.43 ± 3.92 | 0.0004 |
| Red meat other than pork (gram/100 kcal/day) | 0.88 ± 2.21 | 0.94 ± 2.29 | 0.36 |
| Offal (gram/100 kcal/day) | 0.08 ± 0.54 | 0.18 ± 1.04 | <0.0001 |
| Poultry (gram/100 kcal/day) | 1.92 ± 3.38 | 3.05 ± 4.60 | <0.0001 |
| Milk (gram/100 kcal/day) | 8.31 ± 9.88 | 9.37 ± 11.09 | 0.0003 |
| Eggs (gram/100 kcal/day) | 3.16 ± 3.30 | 2.94 ± 3.34 | 0.0164 |
| Fish (gram/100 kcal/day) | 1.96 ± 3.94 | 2.98 ± 4.82 | <0.0001 |
| Fast foods (gram/100 kcal/day) | 0.16 ± 0.91 | 0.15 ± 0.98 | 0.54 |
| Dairy product (gram/100 kcal/day) | 0.02 ± 0.28 | 0.03 ± 0.45 | 0.19 |
| Dessert (gram/100 kcal/day) | 0.70 ± 1.84 | 0.83 ± 1.89 | 0.0113 |
| Candy and sugar (gram/100 kcal/day) | 0.37 ± 1.04 | 0.94 ± 2.09 | <0.0001 |
| Fried foods (gram/100 kcal/day) | 1.00 ± 2.12 | 0.63 ± 1.86 | <0.0001 |
| Catsup and other sources (gram/100 kcal/day) | 0.01 ± 0.19 | 0.05 ± 0.55 | 0.0116 |
| Fungi and algae (gram/100 kcal/day) | 0.45 ± 1.12 | 0.53 ± 1.22 | 0.0106 |
| Sugar-sweetened beverages (gram/100 kcal/day) | 2.06 ± 5.37 | 2.08 ± 5.32 | 0.86 |
| Refined grains (gram/100 kcal/day) | 2.76 ± 3.67 | 6.33 ± 7.15 | <0.0001 |
| Yogurt (gram/100 kcal/day) | 1.12 ± 3.75 | 1.34 ± 4.23 | 0.0496 |

*T-test was used to examine the difference of the intake of other foods between children with high and low wheat intake. P-value<0.0385 was considered statistically significant after controlling the false discovery rate at level of 5% using Benjamin-Hochberg's procedure.

**Table S14. The change in diet score and cardiometabolic risk factors**

|  | Decrease* | Stable | Increase |
| --- | --- | --- | --- |
| Change in HDS | 2140 (37.7) | 2672 (47.1) | 863 (15.2) |
| Change in BMI | 683 (12.2) | 4482 (80) | 441 (7.9) |
| Change in WC | 942 (16.9) | 4006 (71.7) | 641 (11.5) |
| Change in PBF | 1057 (19.4) | 3832 (70.3) | 562 (10.3) |
| Change in SBP | 1127 (20.2) | 3662 (65.5) | 802 (14.3) |
| Change in DBP | 1107 (19.8) | 3735 (66.7) | 756 (13.5) |
| Change in MAP | 1125 (20.1) | 3672 (65.7) | 795 (14.2) |
| Change in TC | 1241 (23.5) | 3426 (65) | 606 (11.5) |
| Change in HDL-C | 1274 (24.2) | 3292 (62.4) | 706 (13.4) |
| Change in LDL-C | 1229 (23.3) | 3351 (63.5) | 695 (13.2) |
| Change in TG | 1290 (24.5) | 3329 (63.1) | 658 (12.5) |
| Change in fasting glucose | 1226 (23.2) | 3415 (64.7) | 636 (12.1) |
| Change in insulin | 1430 (30.8) | 2838 (61.1) | 376 (8.1) |
| Change in HOMA-IR | 1437 (31.0) | 2813 (60.6) | 393 (8.4) |
| Change in CMRS | 1540 (32.0) | 2649 (55.0) | 625 (13.0) |

*Decrease was defined by the change as ≦the mean minus one standard deviation. Stable was defined by the change between the mean minus one standard deviation and the mean plus one standard deviation. The increase was defined by the change as ≧the mean plus one standard deviation.

**Table S15. The change in diet score and cardiometabolic risk factors according to BMI**

|  | BMI at baseline | | |  |
| --- | --- | --- | --- | --- |
|  | Normal  (n=4551) | Overweight  (n=804) | Obesity  (n=628) | P value |
| Change in HDS* | 0.25 ± 1.66 | 0.22 ± 1.63 | 0.09 ± 1.46 | 0.14 |
| Change in BMI | 0.10 ± 0.45 | 0.15 ± 0.74 | -0.28 ± 1.46 | <0.0001 |
| Change in WC | 0.16 ± 0.44 | 0.25 ± 0.65 | 0.18 ± 0.72 | 0.0039 |
| Change in PBF | 0.23 ± 0.73 | 0.15 ± 0.79 | -0.06 ± 1.00 | <0.0001 |
| Change in SBP | 0.01 ± 1.11 | 0.10 ± 1.22 | 0.08 ± 1.28 | 0.0523 |
| Change in DBP | -0.05 ± 1.21 | -0.08 ± 1.36 | -0.11 ± 1.38 | 0.29 |
| Change in MAP | -0.03 ± 1.17 | -0.02 ± 1.31 | -0.04 ± 1.34 | 0.97 |
| Change in TC | 0.01 ± 0.81 | -0.05 ± 0.79 | -0.07 ± 0.88 | 0.0130 |
| Change in HDL-C | 0.61 ± 1.15 | 0.37 ± 1.04 | 0.26 ± 0.94 | <0.0001 |
| Change in LDL-C | 0.27 ± 0.86 | 0.22 ± 0.93 | 0.06 ± 1.00 | 0.0001 |
| Change in TG | 0.01 ± 1.15 | 0.18 ± 1.06 | 0.08 ± 1.12 | 0.0043 |
| Change in fasting glucose | 0.30 ± 1.25 | 0.18 ± 1.25 | 0.16 ± 1.38 | 0.0054 |
| Change in insulin | -0.32 ± 1.57 | -0.25 ± 1.48 | -0.27 ± 1.53 | 0.32 |
| Change in HOMA-IR | -0.25 ± 1.54 | -0.20 ± 1.45 | -0.22 ± 1.51 | 0.47 |
| Change in CMRS | -0.20 ± 2.70 | 0.00 ± 2.66 | -0.27 ± 2.23 | 0.30 |

*The change in diet score and cardiometabolic risk factors was calculated by subtracting the results at baseline from those at follow-up.

**Table S16. Nutrients intake in boys and girls**

|  | All | boys | girls | P-value* |
| --- | --- | --- | --- | --- |
| Energy (kcal/day) | 1273.0 ± 582.8 | 1280.2 ± 587.1 | 1266.8 ± 579.1 | 0.39 |
| Protein intake (g/100 Kcal/day) | 4.33 ± 1.13 | 4.36 ± 1.14 | 4.31 ± 1.12 | 0.0583 |
| Fat intake (g/100 Kcal/day) | 2.96 ± 1.16 | 2.96 ± 1.20 | 2.95 ± 1.11 | 0.67 |
| Carbohydrate intake (g/100 Kcal/day) | 14.17 ± 2.96 | 14.12 ± 3.01 | 14.21 ± 2.89 | 0.28 |
| Fiber intake (g/100 Kcal/day) | 0.53 ± 0.33 | 0.51 ± 0.33 | 0.54 ± 0.32 | 0.0005 |
| Vitamin C intake (mg/100 Kcal/day) | 3.24 ± 2.60 | 3.05 ± 2.54 | 3.43 ± 2.66 | <0.0001 |
| Vitamin E intake (mg/100 Kcal/day) | 0.27 ± 0.19 | 0.26 ± 0.18 | 0.28 ± 0.21 | 0.0001 |
| Carotene intake (ug/100 Kcal/day) | 76.45 ± 85.54 | 72.07 ± 88.11 | 80.56 ± 81.80 | 0.0002 |
| Magnesium intake (mg/100 Kcal/day) | 14.93 ± 3.75 | 14.68 ± 3.70 | 15.18 ± 3.78 | <0.0001 |
| Potassium intake (mg/100 Kcal/day) | 100.15 ± 32.18 | 97.98 ± 31.85 | 102.15 ± 32.25 | <0.0001 |
| Phosphorus intake (mg/100 Kcal/day) | 61.40 ± 13.75 | 61.12 ± 13.64 | 61.63 ± 13.72 | 0.16 |
| Calcium intake (mg/100 Kcal/day) | 30.25 ± 15.80 | 28.78 ± 14.61 | 31.53 ± 16.19 | <0.0001 |
| Iron intake (mg/100 Kcal/day) | 1.20 ± 0.68 | 1.20 ± 0.73 | 1.19 ± 0.63 | 0.71 |

*T-test was used to examine the difference of nutrient intake between boys and girls.

**Table S17. Prevalence of metabolic risk factors and metabolic syndrome associated with healthy diet score**

|  | **Healthy Diet Score** | | | | | | P-trend* |
| --- | --- | --- | --- | --- | --- | --- | --- |
|  | ≤3 | 4 | 5 | 6 | 7 | ≥8 |  |
| Overweight^†^ |  |  |  |  |  |  |  |
| Prevalence | 8.53 | 9.51 | 9.54 | 8.57 | 9.72 | 6.48 |  |
| Odds Ratio (95% CI) | 1.00 | 1.13 (0.79-1.60) | 1.13 (0.82-1.57) | 1.00 (0.72-1.39) | 1.15 (0.81-1.64) | 0.74 (0.45-1.23) | 0.47 |
| High blood pressure |  |  |  |  |  |  |  |
| Prevalence | 7.62 | 9.28 | 10.52 | 10.59 | 10.24 | 8.47 |  |
| Odds Ratio (95% CI) | 1.00 | 1.24 (0.86-1.78) | 1.42 (1.02-1.99) | 1.43 (1.03-2.00) | 1.38 (0.97-1.98) | 1.12 (0.70-1.80) | 0.26 |
| High triglyceride |  |  |  |  |  |  |  |
| Prevalence | 2.75 | 2.96 | 4.52 | 4.38 | 4.91 | 3.42 |  |
| Odds Ratio (95% CI) | 1.00 | 1.08 (0.59-1.98) | 1.67 (0.98-2.86) | 1.62 (0.95-2.75) | 1.82 (1.04-3.19) | 1.25 (0.60-2.63) | 0.12 |
| Low HDL-C |  |  |  |  |  |  |  |
| Prevalence | 5.96 | 7.92 | 5.35 | 5.81 | 4.22 | 4.27 |  |
| Odds Ratio (95% CI) | 1.00 | 1.36 (0.91-2.03) | 0.89 (0.60-1.33) | 0.97 (0.66-1.44) | 0.70 (0.44-1.10) | 0.70 (0.38-1.30) | 0.0203 |
| High fasting glucose |  |  |  |  |  |  |  |
| Prevalence | 2.76 | 2.64 | 1.66 | 2.25 | 2.74 | 1.14 |  |
| Odds Ratio (95% CI) | 1.00 | 0.96 (0.51-1.78) | 0.59 (0.32-1.12) | 0.81 (0.45-1.46) | 0.99 (0.53-1.84) | 0.41 (0.14-1.21) | 0.28 |
| Metabolic syndrome |  |  |  |  |  |  |  |
| Prevalence | 0.77 | 0.66 | 0.98 | 1.18 | 1.27 | 0.86 |  |
| Odds Ratio (95% CI) | 1.00 | 0.86 (0.26-2.84) | 1.29 (0.46-3.62) | 1.54 (0.57-4.20) | 1.67 (0.58-4.82) | 1.13 (0.27-4.74) | 0.77 |

*Logistic regression model was used to examine the association between healthy diet score and cardiometabolic risk factors.

^†^ Overweight was defined by age- and sex-specific waist circumference ≧90^th^ percentile.
